# A national survey of private-sector outpatient care of sick infants and young children in Nepal

**DOI:** 10.1186/s12913-020-05393-1

**Published:** 2020-06-16

**Authors:** Bharat Ban, Stephen Hodgins, Pranita Thapa, Surakschha Thapa, Deepak Joshi, Adhish Dhungana, Anjana KC, Tanya Guenther, Shilu Adhikari, Elaine Scudder, Pavani K. Ram

**Affiliations:** 1 Independent consultant, Kathmandu, Nepal; 2 Save the Children, Washington, D.C., USA; 3 School of Public Health, Univ. of Alberta, Edmonton, Canada; 4 New ERA, Kathmandu, Nepal; 5 USAID’s Maternal & Child Survival Program, Washington, D.C., USA; 6 Dili, Timor-Leste; 7 USAID, Kathmandu, Nepal; 8 UNFPA, Honiara, Solomon Islands; 9 USAID, Washington, D.C., USA; 10 University at Buffalo, Buffalo, New York USA

**Keywords:** Medicine shops, Informal, Village doctors, Private sector, Health markets, Diarrhea, Acute respiratory infection, Possible severe bacterial infection, Antibiotics

## Abstract

**Background:**

Previous research has documented that across South Asia, as well as in some countries in Sub-Saharan Africa, the private sector is the primary source of outpatient care for sick infants and children and, in many settings, informal providers play a bigger role than credentialed health professionals (particularly for the poorer segments of the population). This is the case in Nepal. This study sought to characterize medicine shop-based service providers in rural areas and small urban centers in Nepal, their role in the care and treatment of sick infants and children (with a particular focus on infants aged < 2 months), and the quality of the care provided. A secondary objective was to characterize availability and quality of such care provided by physicians in these settings.

**Methods:**

A nationally representative sample of medicine shops was drawn, in rural settings and small urban centers in Nepal, from 25 of the 75 districts in Nepal, using multi-stage cluster methodology, with a final sample of 501 shops and 82 physician-run clinics. Face-to-face interviews were conducted.

**Results:**

Most medicine shops outside urban areas were not registered with the Department of Drug Administration (DDA). Most functioned as de facto clinics, with credentialed paramedical workers (having 2–3 years of training) diagnosing patients and making treatment decisions. Such a role falls outside their formally sanctioned scope of practice. Quality of care problems were identified among medicine shop-based providers and physicians, including over-use of antibiotics for treating diarrhea, inaccurate weighing technique to determine antibiotic dose, and inappropriate use of injectable steroids for treating potentially severe infections in young infants.

**Conclusions:**

Medicine shop-based practitioners in Nepal represent a particular type of informal provider; although most have recognized paramedical credentials, they offer services falling outside their formal scope of practice. Nevertheless, given the large proportion of the population served by these practitioners, engagement to strengthen quality of care by these providers and referral to the formal health sector is warranted.

## Background

Over the past several decades, a prominent priority of ministries of health in low and middle-income countries (LMIC) and international health agencies has been to reduce child mortality. It has been a notable development success story, with total estimated deaths among children aged less than 5 years declining from 12.6 million in 1990 to 5.4 million in 2017 [1]. Although there have been improvements within all age sub-categories < 5 years, the reduction in deaths has been greatest among children 1–4 years (60% over the period 2000 to 2017), followed by infants aged 1–11 months (51%), with the smallest decline among newborns less than a month old (41%). The two regions that continue to have the highest child mortality rates are South/ South East Asia and Sub-Saharan Africa, and infectious causes continue to make a disproportionate contribution [2]. In Nepal—the setting for this paper—44% of newborn deaths and 88% of deaths among those aged 1–59 months can be attributed to infectious causes [3]. Thus, ensuring timely, appropriate treatment of common infectious illnesses that have the potential to become life-threatening remains an important public health priority.

Outpatient management of childhood illness has received attention in global health over the past two decades, notably through two related initiatives, Integrated Management of Newborn and Child Illness (IMNCI) and Integrated Community Case Management (iCCM). These efforts have primarily focused on improving care within the public sector, particularly at health center and community health worker levels [4]. In most LMICs, the role of the private sector has received much less attention by ministries of health and the global child health movement, although data routinely collected in Demographic and Health Surveys (DHS) and UNICEF’s Multiple Indicator Cluster Surveys (MICS) have long documented that in many Asian and some Sub-Saharan African countries, this care is predominantly sought from the private sector [5]. In many countries, the private sector engaged in sick-child care is—itself—diverse, including qualified physicians (typically catering primarily to urban and financially better-off segments of the population) and a range of non-physician providers, including—at the most informal end of the spectrum—providers with no recognized health worker credentials.

From a multi-country review of data from DHS and MICS surveys [6], a pattern of care-seeking for infant and child illness predominantly from the public sector is evident through much of Sub-Saharan Africa, with some notable exceptions. But in the South and South-East Asian countries reviewed, the private sector was the main source, notably informal providers. Given such evidence for the important role such providers play, there is a sound rationale for seeking to better understand this often-diverse sub-sector, as a basis for elaborating more effective strategies for engaging with it.

### Context

In Nepal, physician-run private clinics are concentrated in urban areas. Private medicine shops (selling both over-the-counter and prescription medications) are found, however, in both urban and rural areas and, as described below, many are staffed by non-physician paramedical workers. There are three such categories of non-physician paramedical workers serving as the main providers of primary healthcare in the public sector in Nepal: Health Assistants (HA), with three academic years of pre-service training, Community Medical Assistants (CMA) and Auxiliary Nurse-Midwives (ANM)—the latter two, with 15–18 months of pre-service training. These categories of health worker staff the most peripheral tier of the government’s primary healthcare system (in rural areas), now designated as health posts. Their scope of practice includes a range of services defined by ministry-issued *Standard Treatment Protocols for Health Posts* [7] for which they are provided an associated set of drugs and other commodities. Within these parameters, these health workers have exercised some degree of autonomy, and have been authorized to dispense what would otherwise be by-prescription-only drugs, without the need for a doctor’s prescription.

Each year over 2500 CMAs and over 1000 new ANMs graduate from a network of public and private sector technical training colleges (accredited under the Council for Technical Education and Vocational Training), along with about 650 HAs [8]. ANMs are licensed under the Nepal Nursing Council; CMAs and HAs, under the Nepal Health Professional Council. Fewer than half of these graduates find employment in the public sector. Most of those not finding public sector employment (at least among the CMAs and HAs) end up working in what are ostensibly just medicine shops.

Analysis of nationally-representative survey data from Nepal [9, 10] has revealed a large and increasing role for the private sector in the treatment of sick infants and young children. Further analysis of data from the 2006 Nepal Demographic and Health Survey (NDHS) [9] revealed that, for a large proportion of cases of child illness for which treatment was sought from medicine shops, someone at the shop examined the child and decided what treatment was required; that is to say these shops function as de facto clinics.

In the 2016 NDHS [10], the private sector (hospitals, clinics and medicine shops) was the main reported source of care for the most recent episode of ARI (74%) and diarrhea (74%) among children below age five. Within the private sector, medicine shops were the most frequently cited source of care, followed by private clinics. From qualitative studies conducted in rural Nepal, caregivers report preferring the private sector (mainly medicine shops) over public sector providers for treatment of childhood illness due to closer access, longer hours of service availability, and perceived better quality of services [11].

According to NDHS 2016 [10], households in the lowest two wealth quintiles rely primarily on medicine shops or pharmacies, and those in the upper three quintiles, on private hospitals or clinics. In the poorest wealth quintile, care-givers reported seeking treatment for their children outside the home for only about one third of cases, with roughly equal proportions using medicine shops/ pharmacies and peripheral government health facilities. Health posts remain an important source of such care for those in the poorest wealth quintile (as well as those living in less-developed hill and mountain areas).

Although private clinics are mainly concentrated in urban areas, medicine shops are found throughout the country; their actual number is unknown, since a large proportion are unregistered. Per policy of the Ministry of Health (MoH) [12], other than over-the-counter products, medicines can only be dispensed by a credentialed pharmacist or pharmacy assistant (having 4 years and 3 years of preservice training, respectively) on the basis of a doctor’s prescription; and all medicine shops are to be registered under the Department of Drug Administration (DDA), with registration renewed each year. However, most medicine shops are not run by pharmacists or pharmacy assistants [13], most drugs are dispensed without a prescription [13], many (perhaps most) medicine shops are not registered with DDA, and only a minority of shops renew their registration annually. As a result, official data provide an inadequate picture of the scale of this sector. This problem has also been noted in studies in India [14, 15].

A recent study (2017), conducted in six districts in Nepal, investigated care of sick young infants in medicine shops and government health posts (see Table 1).

**Table 1:** Six-district study [13]

The study included 60 medicine shops and 24 government health posts, drawn from six districts selected to be representative of the diverse geographies in Nepal. It found that the profile of health worker credentials was essentially the same in medicine shops and government health posts (mainly CMAs, plus other trained paramedical workers) but medicine shop providers, on average, had more years of professional experience. A small proportion of health workers served in both medicine shops and government clinics. Opening hours and availability of health workers were considerably greater in medicine shops than in public health posts, making the shops a more convenient source of care. Approximately half the medicine shops reported, over the previous 3 months, having treated one or more cases of potentially severe infection, among young infants, using injectable antibiotics. By contrast, only three of the 24 public sector health posts reported having treated any such cases. In most respects, there were no differences in quality of care between medicine-shop and health post practitioners. Health workers in medicine shops considered the Ministry of Health a highly credible source for clinical guidelines (and rated pharmaceutical company detailers poorly in this regard) and expressed interest in using such guidelines, if they were made available to them. Similarly, most of those interviewed in private medicine shops indicated interest in participating in a social-franchising network, providing care for sick infants and children, if one were developed.	

The primary objective of the research presented in this paper was to characterize medicine shop-based service providers in Nepal (excluding large metropolitan centers), their role in the outpatient treatment of sick infants and children (particularly young infants), and the quality of the care provided. A secondary objective was to characterize availability and quality of such care provided by private sector physicians.

## Methods

The study employed a multi-stage cluster sample design, intended to be representative nationally (excluding large metropolitan centers). The primary focus was on medicine shop-based practitioners involved in treatment of sick infants and children but the study also included physician-run clinics providing such services. The sample of districts included (25/75) was purposive, aiming for representativeness across geographies and politico-administrative regions; sampling of clusters and of individual service delivery points was done randomly. Data were gathered through face-to-face interviews.

Two particular issues arising when studying sick-child care are more challenging in the private sector [16, 17]. First—unlike similar surveys in the public sector, in settings like Nepal no definitive lists of private sector outlets exist. Second—because services offered by informal sector providers fall in a legal and regulatory grey area, there may be good reason for them to avoid drawing official attention. For investigators interested in this sub-sector, this can make finding such practitioners more difficult and can introduce additional challenges in eliciting accurate, unbiased reports on actual practices, over and above what would be the case for studies of formally-recognized public sector health workers.

### Inclusion criteria

#### Medicine shops

All such shops selling both over-the-counter allopathic medications and those officially classified as by-prescription-only, regardless of DDA registration status; main service provider is not a physician.

#### Private clinics

All outpatient clinics in which a physician is the main service provider or visits the clinic at least 4 days/week; and the physician’s practice includes outpatient care of sick young infants < 2 months of age (note that there are also clinics that may be registered at local level that are staffed only by non-physicians, typically Health Assistants; such clinics were not included in this study).

### Sample size estimation

Sample size determination was driven by the sample required for medicine shop providers treating sick young infants, and by our intention to use this survey as baseline against which to measure change in future surveys. Inputs for the calculation included: design effect 2.0 (taking multi-stage cluster sampling into account), α = 0.05, β = 0.10, an assumed baseline proportion of 0.5 (e.g. for practitioners appropriately providing zinc as part of the treatment for diarrhea), and a minimum change that we wanted to be able to detect of 20 percentage points (i.e. from 0.5 to ≥0.7), and a 95% response rate. Allowing for disaggregated analysis, notably by proximity to a hospital, we arrived at a total of 385 medicine shops providing treatment for sick young infants, which we have rounded up to 400**.**

The sample of medicine shops was to be drawn 16 per district in each of the 25 sampled districts (*n* = 400); in addition, we sought to include 10 private clinics from each of the 10 plains districts and four from each of the 15 sampled hill and mountain districts (*n* = 160).

### Sampling process

**Fig. 1:**
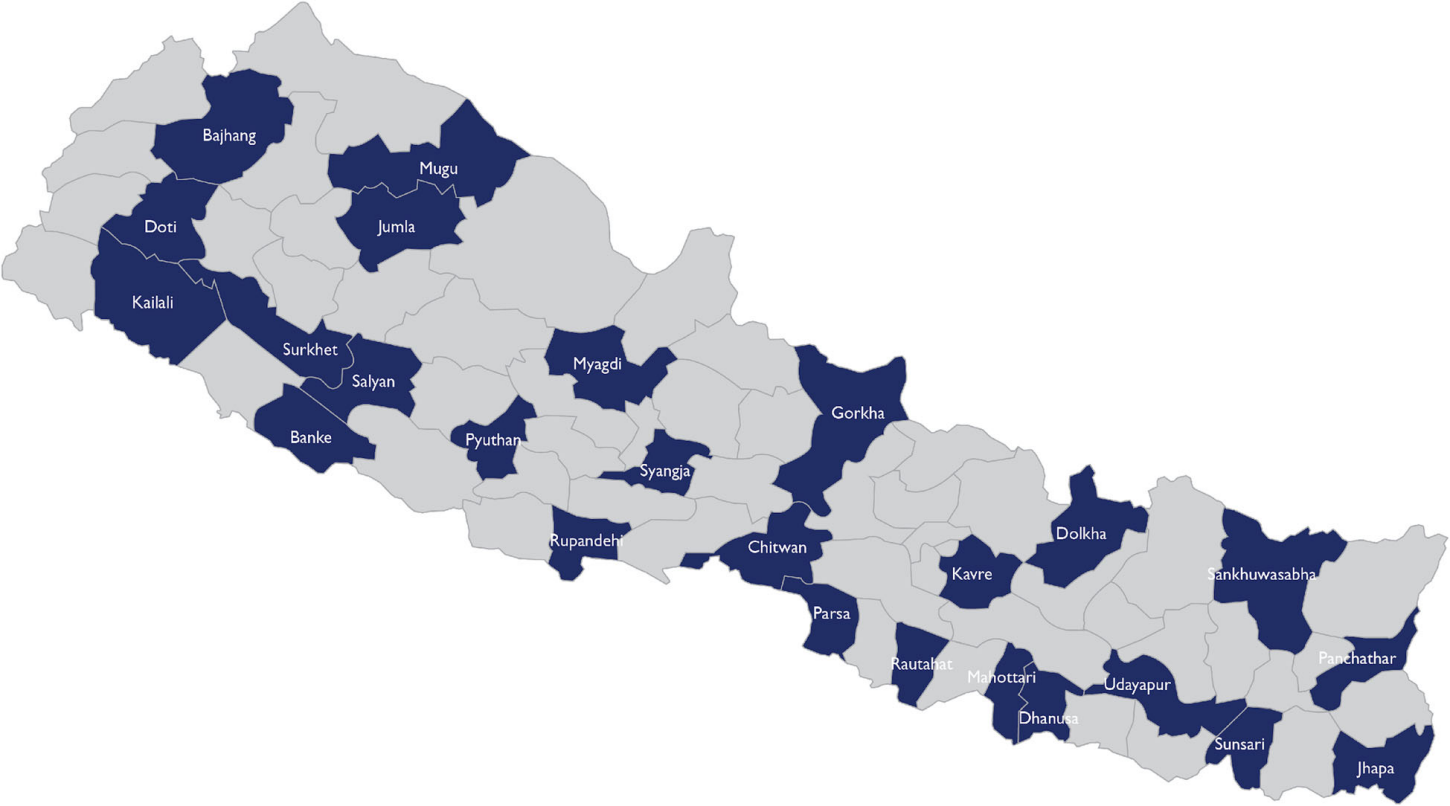
Study districts (figure generated by the study team)

Given the lack of a comprehensive national listing of medicine shops, the process of constructing the sample consisted of three steps:

1. **Selection of districts:** We reviewed an initial national master list of registered medicine shops (from DDA) and then purposively selected 25 survey districts to achieve good representation across all three ecological zones and five administrative regions of the country (see Fig. 1). Several remote mountain districts with very few shops on the national DDA list were excluded, as were Kathmandu and Lalitpur, the two major municipalities making up the Kathmandu metropolitan area, since the complex service delivery environment in this urban setting is radically different from the rest of the country due to the concentration of physicians, private clinics, and hospitals.
2. **Stratification within districts:** Within each survey district, enumeration areas were defined. The team conferred with district public health offices (DPHO) to identify hospitals (public or private), providing in-patient service for infants and children, in some cases including nearby hospitals in adjacent districts (the median number of such hospitals was two in both hill and mountain districts and six in plains districts). Municipalities (urban and rural) within each district were then categorized into three strata based on the time-distance using best available mode of transport to the closest identified hospital (“proximal” < 30 min, “semi-proximal” 30–60 min, and “remote >60 minutes). It was planned that in each of the survey districts, the sample of medicine shops include eight in “proximal”, four in “semi-proximal”, and four in “remote” settings. As the survey was implemented, it became evident in a few districts that not all three strata actually had functioning medicine shops. Notably in three mountain districts, there were no medicine shops within the range of 30–60 min of a hospital, and in two plains districts, there were no medicine shops located > 60 min from a hospital. In such cases, the missing medicine shops were substituted from other districts, comparable with regard to topography. Similarly, when a selected municipality was not found to have any eligible medicine shops, a substitute municipality was randomly selected within the sampled district.
3. **Selection of shops and clinics within clusters:** To form the final sampling frame within selected clusters, the lists of medicine shops were further revised by consulting local key informants from the DPHO, pharmaceutical stockists, local associations of health workers and pharmacies, and local health workers. Shops were drawn randomly from the completed lists, and visited to obtain informed consent, determine eligibility, and assess for their role with regard to treatment of sick young infants. The screening instrument also included questions on services provided for older infants and children with diarrhea or acute respiratory infection. *Non-physician service providers in these medicine shops* who consented and reported at least dispensing medication for sick young infants aged < 2 months, were administered one of the two main survey instruments (and see Table 2, below):
  Tool 1 for those assessing and making treatment decisions for young infants;
  Tool 2 for those reporting only dispensing medication for such cases.
  Note that the survey instruments were developed for this study and have not been published elsewhere. The complete instruments are included as online supplementary files.
  Per the study protocol, our intention was to recruit 10 eligible *physician-run clinics* in each of the 10 plains districts, and four in each of the 15 hill and mountain districts, half from within 30 min of a hospital and half, beyond 30 min (using two strata, not the three used for medicine shops). However, the sample differed from what was planned. It turned out that private clinics were considerably fewer than expected. In six of the 25 survey districts, no private physician-run clinics offering pediatric care were identified, and in most of the other districts very few were found (especially in hill and mountain region). In six of the survey districts where clinics were found, our sample constitutes a complete census of identified clinics having physicians available and willing to be interviewed.

**Table 2:** Variables and survey tools

Main variables included	Survey tools
**Provider profile:** registration status, health worker credentials, sex, age, years of experience treating sick infants and children, also engaged as a health worker in the public sector, number of days/ week and hours/ day services are offered. **Services offered for sick infants and children:** assess and treat sick young infants, older infants and children (vs. only dispense); treat sick young infants using oral antibiotics, or using injectable antibiotics (and reported case volume); **Readiness with regard to sick-child services:** availability of relevant diagnostic equipment, drugs, treatment guidelines. **Quality/ appropriateness of care provided:** what specific signs and symptoms the provider normally checks for, criteria for classifying as “potentially severe”, appropriateness of drugs used, dosing criteria and procedures, follow-up and referral provisions.	**1. Screening/ eligibility module for medicine shops** (included questions on services provided related to diarrhea/ acute respiratory tract infection, among older infants and children). **2. Medicine shop Tool 1**, full instrument, administered to those reporting they had examined and treated or referred sick young infants < 2 months of age over the past 6 months **3. Medicine shop Tool 2**, shorter instrument, administered to those reporting their role with regard to sick infants < 2 months is limited to dispensing **4. Screening/ eligibility module for clinics** **5. Clinic tool**

### Quality of care standards

For assessing quality of care, the following criteria were used, based on Nepal Ministry of Health standards [7]:

- For sick young infants: uses key danger signs to assess severity, antibiotics used per national guidelines (or alternates known to be effective), antibiotic dosing done based on weight assessed using proper technique, desists from dangerous practices (notably, use of injectable steroids), provides assistance for referred cases, follow-up is done
- For diarrhea cases: dispenses ORS and zinc; does not give antibiotics for non-bloody diarrhea; uses ciprofloxacin (or other fluoroquinolone antibiotics) to treat bloody diarrhea
- For ARI cases: uses respiratory rate to assess for possible pneumonia, uses appropriate antibiotics

### Data management and analysis

Data were collected, entered on tablets. Uploaded data were extracted into CSV files and exported to STATA for analysis. In case of any confusion, doubt, or missing data, interviewers revisited and re-interviewed the sample medicine shop or private clinic, before leaving the district.

Analysis was done using STATA, version 12. All analyses were estimated applying sampling weight adjusting for non-response and sample design. To develop sample weights, first, the sampling probability (P) of each medicine shop pertaining to the sampling frame was computed. This was adjusted for the response rate (RR) within that frame. The raw weight (W) was then computed as W = 1/(P X RR), and then standardized by dividing the raw weight of each medical shop by the overall mean of the raw weights of all medical shops, so that the sum of the standardized weights was equal to the overall sample size (number of medical shops).

The study used cluster sampling and stratification based on the ecological region and distance from inpatient-care -providing hospital and, to adjust for their effect, we used complex sample analysis, using the *svyset* command in STATA to generate frequencies. Our analysis consists mainly of proportions, comparing clinics with medicine shops, and disaggregating responses from medicine-shop-based providers by time required to reach the closest hospital. Where comparisons are made in proportions, *p*-values have been calculated, based on chi-square determination.

**Fig. 2:**
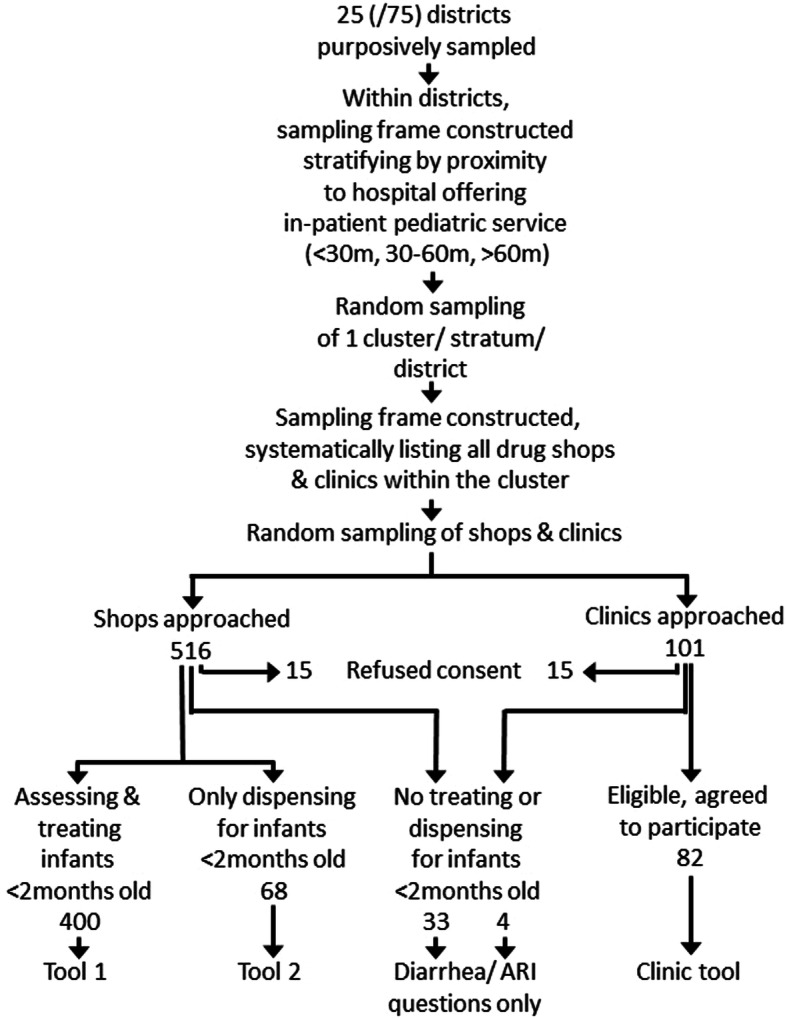
Flow Diagram

Study participants were recruited as illustrated in the Flow Diagram (see Fig. 2), yielding (per design) 400 medicine shops treating infants < 2 months of age, 68 dispensing for such cases but not involved in assessment and treatment decision-making, and 33 not providing any services for young infants but potentially providing services for sick older infants and children. The sample also included 82 clinics involved in treatment of sick young infants, and four not involved in young infant care.

## Results

### Service provider characteristics

Most medicine shops in urban settings, close to a hospital, were registered with the DDA; most of those more than 30 min away from a hospital were not (*p* < .001) (see Table 3). Over three quarters of shops were staffed by individuals reporting at least two academic years of professional paramedical training (69% reported having credentials as certified medical assistants or health assistants). Pharmacists/ pharmacy assistants were more common in urban medical shops, close to a hospital (10%, vs. 6% in more distant shops, *p* = 0.13). Fifteen percent of medicine-shop providers also worked in public sector health facilities; among physicians, the proportion was more than twice as high (34%; *p* = .002 on the difference). Almost all medicine shops reported having services available 7 days a week (95%, vs 83% of clinic-based physicians, *p* = 0.002) and being open at least 11 h a day (93%, vs. 85% of physicians, *p* = 0.1). A small proportion of shops (10%) reported that physicians were sometimes available at the shop to see patients; for those that did, typically this was once or twice a week (data available on request).

**Table 3:** Profile of medicine shops & private clinics, engaged in treatment of young infants

	Medicine shops (%)				Physician-run clinics (%)
Proximity to hospital with in-patient pediatrics service (in minutes)	< 30 min *n* = 200	30–60 min *n* = 100	>60 min *n* = 100	All *n* = 400	*n* = 82
DDA registration	74	36	34	55	N/A
**Professional credentials**:					
Pediatrician					38
Other physician					62
CMA/ HA	66	74	72	69	
Pharmacist/ Pharmacy Assistants	10	6	5	8	
ANM/ nurse	6	5	7	6	
Other paramedical	1	2	6	3	
No professional training	17	12	10	14	
Sex (% male)	86	94	79	86	98*
**Age** - mean				38 years	38 years
< 30 years	22	20	30	24	17
30 to 40 years	44	52	43	45	57
> 40 years	34	28	27	31	26
10+ yrs. experience treating sick infants	53	34	43	46	31†
Dual practice in public sector H facility	13	17	19	15	34*
Also does in-patient pediatrics					62
**Services available:**					
11h hours/ day	94	88	97	93	85
7 days/ week	98	96	90	95	83*
Physician on site at least once/ week	9	5	11	9	N/A

Medicine shop data presented in this table are restricted to those to which the full survey instrument (Tool 1) was administered (*n* = 400); shops that reported only dispensing medicines for young infant illness and not involved in assessment and treatment decisions are not included here

*Acronyms*: *DDA* Department of Drug Administration, *CMA* Certified Medical Assistant, *HA* Health Assistant, *ANM* Auxiliary Nurse-Midwife, *H facility* health facility, *N/A* not applicable

* *p*-value on difference between medicine shops and clinics < 0.01

† *p*-value on difference between medicine shops and clinics < 0.05

### Infant and child illness-related services offered

Among medicine shop-based practitioners who reported treating infants < 2 months of age, most (56%) reported having treated no more than 20 cases with oral antibiotics over the previous 6 months. A relatively small proportion (10%) reported much higher volumes, over 100 cases. The volume of such cases reported by physicians was, on average, considerably higher (data available on request).

Although only a comparatively small proportion of medicine shop practitioners (19%) reported having used parenteral antibiotics for treating young infants over the past 6 months, the proportion was considerably higher among those based more than an hour away from a hospital (33%) compared to those within 30 min (13%, *p* = .027 on the difference). Almost half of the physicians in the sample reported having used parenteral antibiotics for at least some of their young infant patients (46%), although the proportion reporting such treatment for infants < 1 month of age was smaller (36%).

By design, as indicated earlier, the survey was to include 400 medicine shop-based practitioners who reported treating infants < 2 months of age. This practice was determined through use of a screening instrument. In administering the instrument:

- an additional 68 were identified, who reported only dispensing for infants of this age (not assessing and treating)—they were administered a somewhat shorter survey instrument (Tool 2); and
- a further 33, who reported no treatment or dispensing for young infants.

All of those administered the screening instrument (*n* = 501) were asked a small set of questions concerning services for illness among older infants and children, aged two to 59 months. As seen in Table 4, a large proportion reported that they were involved in assessing and treating diarrhea (87%) and acute respiratory infections (86%) in this age group, although the proportion reporting such a service was smaller among shops close to hospitals (79%) than those more distant (for diarrhea, *p*-value on the difference < 0.001; for ARI, *p*-value = 0.007).

**Table 4:** Medicine Shops Treating Diarrhea & ARI, ages 2–59 months, vs. only Dispensing

Proximity to hospital (minutes)	< 30 min (%) *n* = 279	30–60 min (%) *n* = 114	>60 min (%) *n* = 108	All (%) *n* = 501
Diarrhea	79	98	95	87
Acute respiratory infection	79	95	94	86

### Readiness for treatment of sick young infants (*n* = 400 medicine shops, *N* = 82 clinics)

Only 15% of medicine shops providing treatment for sick young infants had a scale suitable for weighing young infants vs. 72% of medical clinics (*p* < 0.001); 74% had an adult scale vs. 95% of clinics (*p* < 0.001). (By comparison, in a nationally representative survey of public sector health facilities—the 2015 Nepal Health Facility Survey (NHFS)—infant scales were found in 65% of health posts [18]). Digital thermometers were present in 94% of medicine shops and 98% of clinics (and 94% of health posts in the NHFS). Similarly, stethoscopes were present in 97% of medicine shops and 100% of clinics (and 100% of health posts, NHFS), and 94% of medicine shop-based practitioners had a cell phone (vs. 100% of physicians). Eighty-five percent of medicine shops had a timer or watch available for counting respiratory rate, vs. 99% of clinics (95% of health posts in NHFS). Pulse oximeters were present in 16% of medicine shops and 76% of clinics.

Commodities found to be available at the time of the survey in almost all medicine shops included ORS sachets (98%, although only in 88% among shops in mountain districts, *p* = 0.013), pediatric formulations of amoxicillin (97%), and cefixime (93%). Zinc was available in over three quarters of medicine shops in hill and plains districts but only 43% of shops in mountain districts (74% in the sample, as a whole; *p*-value on the difference < 0.001). Cotrimoxazole suspension or dispersible tablets were somewhat less widely available: 62% of shops in plains, 57% hill, and 49% of mountain districts. Injectable antibiotics suitable for treating pneumonia or sepsis were available in fewer of the shops (ceftriaxone 55%, gentamicin 40%, cefotaxime 37%, and ampicillin 24%).

Just over a quarter of medicine shop-based practitioners (27%) reported having ever having received IMNCI training, with a higher proportion among those within 30 min of a hospital (33%, *p*-value on the difference = 0.24). Half of surveyed physicians reported having received this training (49%, *p* = 0.013 on the difference with medicine-shop providers).

Reference materials present on the treatment of sick infants and children included: current index of medical specialties (42% of medicine shops, 40% of clinics), treatment guidelines for the government’s IMNCI program (25% of medicine shops, 50% of clinics (and 67% of health posts in NHFS [18])), and course books (8% of medicine shops, 24% of clinics). Registers were present and used for recording information on cases of sick infants by 12% of medicine shops and 57% of clinics

### Quality of care for sick young infants

A variety of open-ended questions (without specific prompts) were asked related to quality or appropriateness of care for sick young infants. For several potential danger signs, fewer medicine shop practitioners than physicians reported specifically considering them when assessing sick young infants, notably: level of consciousness, umbilical redness or pus, and seizures or feeding problems as reported by the care-giver (see Table 5). The survey also included an unprompted question on what assessment findings would suggest that a sick young infant may have a potentially severe infection. Most medicine shop providers responded that high temperature (89%), severe chest in-drawing (82%), and rapid respiratory rate (86%) would be danger signs. Other important potential danger signs that were less frequently mentioned included: abnormally low temperature (38%), care-giver report of poor feeding (29%) or seizures (20%), and moving only when stimulated (17%) or unconscious (14%). A larger proportion of clinic-based physicians were able to cite at least four recognized dangers signs of possible severe infection (81%), than medicine shop-based practitioners (66%) although because the questions were asked slightly differently, we have not calculated a *p*-value on this apparent difference.

**Table 5:** Quality/ Appropriateness of Care for Sick Young Infants (unprompted questions)

	Medicine shops (%)				Physician-run Clinics (%)
Proximity to hospital (in minutes)	< 30 min	30–60 min	>60 min	All	
Sick young infants < 2 months of age	*n* = 200	*n* = 100	*n* = 100	***n*** **= 400**	***n*** **= 82**
**Reports assessing for:**					
Respiratory rate	92	92	86	**91**	**90**
Temperature	91	80	90	**88**	**89**
Feeding (as reported by mother)	67	65	61	**65**	**82**†
Seizures (as reported by mother)	17	36	23	**24**	**62**†
Weight	28	44	29	**32**	**66**†
Chest in-drawing	48	59	57	**53**	**60**
Umbilical redness or pus	31	25	24	**28**	**48**††
Level of consciousness	14	21	23	**18**	**46**†
**Treatment**					
**Usual first-line*oral*antibiotic**	*n* = 200	*n* = 100	*n* = 100	***n*** **= 400**	***n*** **= 82**
Amoxicillin (+/− clavulanate)	63	76	77	**73**	**82**
Cefixime	41	30	28	**35**	**39**
Cefpodoxime	1	7	2	**3**	**17**
Cotrimoxazole	6	13	8	**8**	**8**
others	7	8	18	**10**	**8**
**Usual first-line*injectable*antibiotic**^**a**^	*n* = 28	*n* = 17	*n* = 36	***n*** **= 81**	***N*** **= 38**
Gentamicin	51	34	63	**53**	**21**††
Ampicillin	16	9	16	**14**	**37**††
Cefotaxime	20	47	16	**24**	**29**
Ceftriaxone	20	17	22	**20**	**24**
Amikacin	9	20	0	**7**	**16**
others	0	0	3	**1**	**11**††
**Other treatments used**	*n* = 200	*n* = 100	*n* = 100	***n*** **= 400**	***N*** **= 82**
Bronchodilators	50	37	34	**43**	**43**
Injectable steroids	11	11	11	**11**	**21**
Steroids given within past 6 mo.	5	10	7	**6**	**12**
Dosage determination & weighing	*n* = 200	*n* = 100	*n* = 100	***n*** **= 400**	***N*** **= 82**
Determines dose by age, not weight	30	30	50	**35**	**10**†
**Doses by weight, determined by**^**b**^**:**	*n* = 140	*n* = 68	*n* = 50	***n*** **= 258**	***N*** **= 71**
Salter or pan scale	9	9	12	**10**	**63**
Adult scale (subtract. technique)	82	81	68	**79**	**37**
Estimates by looking	9	10	20	**11**	**0**
Among those weighed,	*n* = 129	*n* = 61	*n* = 39	***n*** **= 229**	***N*** **= 71**
leaves baby’s clothes on	97	97	100	**97**	**80**††
Shortened treatment course	*n* = 200	*n* = 100	*n* = 100	***n*** **= 400**	***N*** **= 82**
Somewhat or very often	50	53	36	**48**	**40**
**Danger sign referral**	*n* = 200	*n* = 100	*n* = 100	***n*** **= 400**	***N*** **= 82**
Helps arrange transport	60	83	57	**65**	**59**
Provides referral note	42	65	58	**52**	**77**†
Calls ahead to MD at receiving HF	25	16	20	**22**	**39**††
Gives pre-referral oral antibiotics	42	60	59	**51**	**48**
Gives pre-referral inj. Antibiotics	5	8	13	**8**	**12**
Schedules follow-up visits	99	98	99	**99**	**95**

^a^ denominator includes only those reporting having used injectable antibiotics to treat sick infants over the previous 6 months

^b^ denominator includes only those reporting determining dose based on weight

† *p*-value on difference between medicine shops and clinics < 0.001

†† *p*-value on difference between medicine shops and clinics < 0.05

Most medicine shop providers and physicians reported using amoxicillin as first-line oral antibiotic for these cases (in line with national guidelines), followed by cefixime (see Table 6, below). Choice of first-line injectable antibiotics appeared to differ, with physicians reporting ampicillin (37%) and cefotazime (29%) or ceftriaxone (24%); and medicine shop providers reporting gentamicin (53%), although in both cases the samples were small.

**Table 6:** Quality of Treatment for Diarrhea & ARI, among Infants/ Children 2-59 m

	Medicine shops (%)				Physician-run Clinics (%)
Proximity to hospital (in minutes)	< 30 min	30–60 min	>60 min	All	
**Diarrhea**					
Dispense or refer only	57	2	5	**64**	
Assess & treat	*n* = 222	*n* = 112	*n* = 103	***n*** **= 437**	***n*** **= 86**
ORS most/ all cases	93	89	88	**91**	**98**
Zinc most/ all cases	69	61	67	**66**	**90**†
No antibiotics for non-bloody diarrhea	25	23	22	**24**	**30**
*Antibiotics for bloody diarrhea:*					
Ciprofloxacin or other quinolone	19	40	32	**32**	**18**††
Metronidazole	26	28	29	**27**	**31**
A cephalosporin antibiotic	16	11	11	**14**	**38**†
Cotrimoxozole	22	18	23	**21**	**11**
**Acute Respiratory Infection**					
Dispense/ refer only	59	5	6	**70**	
Assess & treat	*n* = 220	*n* = 109	*n* = 102	***n*** **= 431**	***n*** **= 86**
Antibiotic based on respiratory rate	97	99	98	**98**	**98**
*Specific antibiotics used:*					
Amoxicillin +/− clavulanate	70	68	70	**69**	**65**
Cefixime	17	23	16	**18**	**12**
Other cephalosporin	3	4	2	**3**	**9**
Cotrimoxazole	8	5	8	**7**	**2**
Azithromicin	0	0	0	**0**	**7**
Other	3	1	5	**3**	**5**

Note that due to rounding, in some instances totals may not sum to exactly 100%

† *p*-value on difference between medicine shops and clinics < 0.001

†† *p*-value on difference between medicine shops and clinics < 0.05

The same proportion of medicine shops and clinics reported commonly using bronchodilators for treating sick young infants (43%). Eleven percent of medicine-shop providers reported at least some use of injectable steroids for treating sick young infants; 6% within the past 6 months (with no differences in proportion across proximity strata); 21% of physicians reported at least some use of steroids, 12% over the previous 6 months (for the difference between medicine shops and clinics in any reported use of steroids, *p*-value = 0.013).

Over one third of medicine shop providers (35%) reported determining antibiotic dosing for young infants based on age, not weight; this was less common among physicians (10%, *p*-value on difference < 0.001). Of those who reported determining dosage based on weight, only 10% of medicine shops reported using a suitable scale (Salter or pan) vs. 63%, among physicians (*p*-value < 0.001). Over three quarters of medicine shop providers (79%) used a technique that entailed weighing the mother with and without the baby and subtracting to determine the baby’s weight. This was also commonly done by physicians (37%, *p*-value < 0.001). Almost all medicine shop practitioners (97%) reported that they do not normally remove the baby’s clothing/ coverings to do the weighing; similarly, 80% of physicians also reported not removing the baby’s clothing/ coverings (*p*-value on the difference = 0.005). Such practices compromise accuracy of antibiotic dosing (and safety, notably for aminoglycoside antibiotics).

Giving an abbreviated course of antibiotics was done at least somewhat often by close to half of medicine shop providers (48%) and physicians (40%). A significant proportion of both medicine shop providers and physicians reported commonly helping arrange transport for referred cases (65 and 59%, respectively, *p*-value = 0.37) and providing a referral note (52 and 77%, respectively; *p*-value = 0.001). Only 22% of medicine shop providers reported calling the physician at the receiving institution (vs. 39% of the physicians, p-value on the difference = 0.02).

### Quality of care for older infants and children up to 59 months

For diarrhea, most medicine shops were doing more than dispensing medicines; 87% reported assessing and making treatment decisions (97% of those located ≥30 min from a hospital, vs. 79% among those < 30 min, *p* < 0.001). In Table 6, we present reported practices restricted to those doing more than dispensing (note that for this analysis, 37 medicine shops not reporting any treatment of infants < 2 months were included). Dispensing oral rehydration solution (ORS) and zinc—over-the-counter products—falls within their legally permitted scope of practice. Overall, the likelihood of appropriate treatment for diarrhea was lower in medicine shops than in physician-run clinics. The proportion who reported *prescribing ORS most or all of the time* was 91% (vs. 98% of clinics, *p*-value = 0.06); 66% of medicine shops reported *routinely using zinc* (vs. 90% of clinics, p-value on the difference < 0.001). A small proportion of providers reported *routinely using antibiotics to treat diarrhea* (6% of medicine shops, 4% of physician-run clinics), however 76% of medicine shop practitioners and 70% of physicians reported at least some use of antibiotics for non-bloody diarrhea (*p*-value on the difference = 0.13). Metronidazole was the most commonly used antibiotic for non-bloody diarrhea (medicine shops 75%, clinics 69%, *p* = 0.22). Other than for specific clinical presentations, e.g. suggestive of giardiasis, this is an inappropriate antibiotic for diarrhea.

For bloody diarrhea, one third of medicine shop practitioners (32%) reported routinely giving ciprofloxacin or other fluoroquinolone antibiotics as first line treatment (in line with IMNCI guidelines) but only 18% of clinics (p-value on the difference = 0.016). Metronidazole was commonly used (reported by 27% of medicine shops, 31% of clinics, *p*-value 0.32); oral cephalosporins were reported by 38% of clinic-based practitioners as first line. Neither are considered appropriate treatment.

For ARI (as with diarrhea case management), the overwhelming majority of medicine shops (86%) reported not just dispensing treatment but also assessing and making treatment decisions. Among semi-proximal and remotely located medicine shops, 94% reported assessing, making treatment decisions, and dispensing. Since this entails dispensing of antibiotics without a physician’s prescription, this lies outside the formally recognized scope of practice for non-physicians working in the private sector.

Virtually all providers reported using respiratory rate to classify ARI cases for antibiotic treatment. Amoxicillin (+/− clavulanate) was reported as first-line treatment by most providers in medicine shops (69%) and physician-run clinics (65%), consistent with the government’s IMNCI recommended treatment. Cefixime was the second most often reported first-line antibiotic (18% of medicine shops, 12% of clinics). Although not the recommended first-line drug, this is also an efficacious treatment for ARI. Overwhelmingly, providers reported using syrup/ suspension formulations for treating young children (96%), not dispersible tablets (which typically are used in public-sector programs).

## Discussion

### Key findings

This study has documented that in rural areas and smaller urban centers in Nepal, private, physician-run, outpatient clinics caring for sick infants and young children are few and far between, particularly in hill and mountain areas where half the population lives, such that we were not able to recruit the planned number of clinics in the survey. By contrast, medicine shops are much more widely distributed and easily accessible to the population. By our estimate,^1^ there are approximately 4–5 times more medicine shops than government health posts.

Our study results indicate that, in Nepal, most medicine shops are run by individuals who report having formal credentials as paramedical workers (mainly certified medical assistants and health assistants). The study also found that most medicine shops function as de facto clinics (though the proportion is lower in urban settings, close to hospitals), where paramedical workers examine patients and make treatment decisions, generally charging only for sale of medications. Although such workers are permitted a reasonably broad scope of practice if they are working in government health posts or primary healthcare centers, such a role is not permitted to them in private practice.

Dual practice was relatively common in our sample: 15% of medicine shop-based practitioners and 34% of physicians reported also working in public sector health facilities.

The study documented certain deficiencies in care provided, as reported by both physicians and medicine shop-based practitioners. On some measures, physicians performed better than medicine-shop providers, for example physicians appear to have a better understanding of potential danger signs in sick young infants. The study highlighted several areas of concern:

- There were problems with procedures used to determine antibiotic dosing for sick young infants; this is potentially dangerous, especially for aminoglycoside antibiotics, which have dose-related toxicity risks.
- Antibiotics were widely used for treating child diarrhea, and the most-used antibiotic products are inappropriate (metronidazole, while suitable for specific clinical syndromes—for example for illness suggesting giardiasis, is not effective against the main pathogens responsible for bloody diarrhea).
- Although most medicine shops and physicians reported not using injectable steroids for treating sick young infants, a minority did report such use; probably in almost all instances this would have been inappropriate and potentially dangerous.

### Limitations

Selection of districts was done purposively to achieve an even distribution of districts across the three ecological zones and five administrative regions. Since this was not done randomly, the findings cannot be considered to provide point estimates that are statistically representative of the country as a whole. Furthermore, the district sample excluded Kathmandu and Lalitpur districts, covering the Kathmandu metropolitan area. Thus, study findings do not provide insights into the role of medicine shops in this metropolitan setting.

Selection of clusters, and of medicine shops within clusters, was done randomly. However, in each of the 25 districts surveyed only one cluster was selected for each of the three proximity strata. In comparison to a sampling strategy with a larger number of clusters drawn per district, this can be expected to reduce sample diversity, resulting in data clustering, which reduces statistical power.

During fieldwork it was determined that private physician-run clinics providing outpatient pediatric care were considerably fewer than expected. In a quarter of the districts sampled, no such clinics were identified at all, and in most other districts in the sample there were too few found to allow us to include one clinic per proximal cluster and another from semi-proximal or remote clusters, as specified in the study protocol. The overall size of the clinic sample ended up only half of what was planned. The improvisational nature of actual drawing of the sample in the field and the small sample size limit conclusions than can be drawn about these providers.

Given that most of the medicine shops surveyed are evidently engaged in practices not sanctioned by the government regulatory body responsible for this sector, response bias is an important potential threat to validity of findings on such practices. We asked medicine shop-based practitioners: “What is your highest academic qualification, related to medical care?” Some participants may have over-reported their qualifications. One might also expect under-reporting of certain treatment practices and dispensing of by-prescription-only medications without a prescription.

Finally, one important objective of the study was to investigate quality and appropriateness of care for infant and child illness. However, our data were based entirely on self-report by service provider, not on review of case records, simulated clients or observing actual care. In addition to potential response bias, a consequence of this strategy is that the range of dimensions of quality that could be investigated was limited. For example, we are not in a position to comment on how appropriately providers differentiated between cases of uncomplicated upper respiratory tract infection—for which antibiotics would generally not be indicated—and cases where there is a reasonable suspicion of pneumonia.

We propose that within the constraints just noted, the results of our study do generalize across Nepal (excluding major metropolitan areas). Although informal, non-physician, private-sector providers also play an important role in the care of sick infants and children elsewhere in South Asia [19] and—in many instances—do so based in medicine shops [20], it would not be valid to conclude from our study results that the professional profile we have documented in Nepal would be found in other South Asian settings. Furthermore, although specific issues with quality of care identified in this study (e.g. overuse of antibiotics for treating diarrhea) would likely also be found in other South Asian settings, the relative importance of such problems is likely to vary by setting and by sub-type of provider.

The one other country in South Asia in which an analogous national survey of medicine shop providers has been conducted is Bangladesh [20]. As we have noted, Nepal has a well-established peripheral-level government primary healthcare structure—the health post—staffed by professionalized paramedical workers with at least 15–18 months of preservice training; it is these same categories of workers who appear to run most medicine shops in Nepal. In Bangladesh, medicine shop-based practitioners are primarily “village doctors,” a small proportion of whom have received a government-sponsored 12-month training as “Palli Chikitsok”; most have only a few weeks or months of training from a semi-formal private institution [20]. Both Nepal’s medicine shop-based practitioners and Bangladesh’s “village doctors”—though based in medicine shops and employing a business model relying primarily on sales rather than consultation fees—are in effect running outpatient clinics. As we have noted, medicine shop-based practitioners in Nepal largely have credentials equivalent to, and offer quality of care similar to, their counterparts working in government health posts. Similarly, Bangladeshi “village doctors” appear, on average, to have somewhat similar durations of pre-service training to those working in government Community Healthcare Centers (which are somewhat analogous to Nepal’s health posts, though of more recent origin), but this is considerably shorter than their Nepali counterparts. In both Nepal and Bangladesh, medicine shop-based practitioners are responsible for a large proportion of outpatient care of sick infants and newborns, but in neither case is this role officially sanctioned (particularly when it involves dispensing antibiotics without prescription). So, there are different versions of “informality” for outpatient services [20, 21]. In Bangladesh, we could consider both the *practice* (operating a de facto clinic out of a medicine shop) and the *cadre* (“village doctor”) as informal, or not formally recognized. In Nepal, the practice of operating de facto clinics out of medicine shops is certainly not formally recognized by government, but those providing the service generally have recognized credentials that would allow them a similar scope of practice, if based in a government health post. Elsewhere in different parts of South Asia we would see different variations on informality, each with its own implications for quality of care and potential for engagement from ministries of health.

### Policy and program implications

Across settings where informal, non-physician practitioners are responsible for a substantial proportion of care for infant and childhood illness, particularly for poorer segments of the population, in most instances it will be neither feasible (given relatively weak enforcement capacity in most of these settings) nor in the public interest to simply try to shut these practitioners down [22, 23]. But, if not enforcement, then what? One comparatively simple step would be to make Ministry of Health standard treatment protocols and guidelines more easily available. In the earlier 6-district study [13], investigators found that quality of care for infant and childhood illness is similar between government health posts and medicine shops; furthermore, medicine shop-based practitioners considered the Ministry of Health a highly credible source of treatment information and expressed interest in having access to treatment guidelines. But, as pointed out by Montagu and Goodman [24], training or provision of treatment information is premised on the assumption that the key constraint preventing appropriate care is lack of knowledge. That may be warranted for some practices and for some categories of informal health workers but for many, more comprehensive approaches will be needed to improve care practices.

A range of strategies has been used to address quality of care in such private outlets. There are many examples of large-scale “social marketing” programs that make branded public health commodities available through private medicine shops, often on a subsidized basis. Relevant to treatment of childhood illness, this has been a common strategy for increasing use of ORS for treatment of childhood diarrhea. Beyond distribution of specific commodities, programs have engaged medicine shops seeking to improve quality of specific health services, using a “social franchising” strategy that entails private outlets joining a network, being allowed to use the network branding, and often benefiting from training, marketing, and quality assurance provided by the franchisor [25, 26]. Nepal has a long history with both social marketing and social franchising. Working under the oversight of the Ministry of Health, a local donor-supported, not-for-profit entity has distributed commodities (some, subsidized) and, since 1994, has developed and supported a network of medicine shops (now numbering over 3000), under the brand *Sangini*, that distribute contraceptive products. Beyond simply dispensing, they are authorized to initiate women on contraception and to administer injectable medroxyprogesterone acetate. Support from the franchisor has included initial training and modest ongoing supervision and quality assurance, costs of which have been covered by the donor. So, Nepal has a well-established and long-running family planning social franchising program, under Ministry oversight, that can serve as a model for other technical areas. Those leading such efforts in Nepal can also draw lessons from Bangladesh experience engaging with village doctors to improve care of sick children [27, 28].

Note that a technical report is available on this survey [29], with further detail on methods and additional analyses not included in this paper.

## Conclusions

Although generally not with official recognition, medical shops are an important source of outpatient care—especially for the poor—across South Asia and in several African countries (Nigeria being a notable example). Nepal offers a particular type of such informal practice: most medicine shops are run by graduates of accredited training colleges, with two or three years of preservice paramedical training, and have registered with national licensing bodies. They are assessing and treating patients; in many instances, this may be largely in conformity with the Ministry’s *Standard Treatment Protocols for Health Posts*. However, the scope of practice defined by these treatment protocols only covers health workers based in government health facilities; there is no provision for them to play such a role in the private sector. Our study documented deficiencies in quality for both medicine shop-based practitioners and private physicians but, as documented in nationally representative household surveys [9, 10], medicine shop providers are clearly filling an important need. Currently, efforts by major global health actors addressing outpatient care of sick infants and children focus almost exclusively on peripheral-level government primary healthcare services and community health workers, under the rubric of IMNCI and iCCM, although there is a growing recognition of the need to engage private providers [21, 30, 31]. In settings like Nepal, most such care is actually provided by informal or semi-informal private practitioners. If our interest is improved access to and quality of such care, and better health outcomes at population scale, we need to figure out how best to constructively engage these practitioners.

## Key concluding comments

- In Nepal, most medicine shops function as de facto clinics, with regard to treatment of childhood illness.
- Although this role is not officially sanctioned, they serve a valuable function, in particular for the rural poor.
- In the case of Nepal, most medicine-shop service providers in fact have credentials equivalent to those providing similar services, based in government health posts
- Although findings of this study do not generalize directly to other countries, they point to analogous phenomena elsewhere in the region.
- In efforts to improve outcomes for childhood illness in low- and low middle-income countries, the role of private-sector informal providers should not continue to be ignored.

## Supplementary information

**Additional file 1.**

## Data Availability

The dataset used in this study is available from the corresponding author on reasonable request.

## Abbreviations

ANM: Auxiliary Nurse Midwife
ARI: Acute Respiratory Infection
CAPI: Computer-Assisted Personal Interviewing
CHCP: Community Health Care Provider
CMA: Certified Medical Assistant
DDA: Department of Drug Administration
DHS: Demographic and Health Survey
DPHO: District Public Health Office
FWA: Family Welfare Assistant
HA: Health Assistant
HF: Health facility
iCCM: Integrated Community Case Management
IMNCI: Integrated Management of Neonatal and Childhood Illness
MICS: Multiple Indicator Cluster Survey
NDHS: Nepal Demographic and Heath Survey
NHFS: National Health Facility Survey
ORS: Oral Rehydration Solution

## Footnotes

1 Outside of urban areas, most medicine shops are not registered with the drug regulatory agency, DDA. If we assume that the proportion of medicine shops in the survey that reported being registered (57%) is a good approximation of the proportion in Nepal, outside Kathmandu valley, and take into account that there are approximately 10,000 shops outside Kathmandu valley licensed by DDA to sell allopathic, human products, we would expect approximately 7500 additional *unregistered* shops, for a total in the range of 15–20,000 (outside Kathmandu), which is 4–5 times the number of government health posts in the country.
