# Supplementary material for: A national survey of private-sector outpatient care of sick infants and young children in Nepal
Source: BMC Health Serv Res. 2020 Jun 16;20:545. doi: 10.1186/s12913-020-05393-1 (PMC7298835; doi:10.1186/s12913-020-05393-1)
Supplement: Supplementary file 1 — Additional file 1. [file 12913_2020_5393_MOESM1_ESM.zip › Pvt_Clinic_survey instrumentR4.docx]

**A National Survey on Care of Possible Serious Bacterial Infection (PSBI) among Sick Young Infants 0-2 months in Private Sector Medical Shops and Clinics in Nepal**

**(Physician operated Private Clinics)**

**Screening questionnaire to identify private clinics that treat sick young infants 0-2 months with antibiotics**

***Note: Ask to speak to the main person responsible for treating sick young infants. If they are not available on first attempt, schedule a time to return when they will be able to complete the interview.***

**General Information**

| **District Code** | |  |
| --- | --- | --- |
| **VDC/Municipality Name and Code:** ____________________ | |  |
| **Name of Palika** | |  |
| **Ward No**. | |  |
| **Name of Private Clinic:** __________________________________  **Questionnaire No……………………………………………………………………**  | | |
| **Type of Clinic** | Outpatient only ………….1  Outpatient and inpatient …2  Other (Specify) _________________________96 | |
| **Type of Strata (refer to the sampling)** | Proximal (half an hour distance from hospital) 1  Semi Proximal (30 minutes to 1 hour) 2  Distant (more than 1 hour) 3 | |
| **Interviewer code…………………..…** |  | |
| **Date of Screening (A.D)……………….** |     Day Month Year | |
| **GPS (Latitude)** ………………….………. |  .  | |
| **GPS (Longitude)**………………….……… |  .  | |
| **Survey type**………………….…………… | National  | |

**Questions about management of diarrhea and respiratory infections among young children aged 2-59 months**

| ***Administer Consent Form***  **Consent Given…………………… Start the interview **  **Consent Not Given……………… Thank the Respondent and END the interview ** |
| --- |

**400. Now I would like to ask you a few questions about the assessment and treatment of children aged 2-59 months.**

| **S.No.** | **Questions** | **Responses** | | | | **Go To** |
| --- | --- | --- | --- | --- | --- | --- |
| 401 | a) For cases of diarrhea, how often do you give ORS; most or all the time, sometimes, but less than half of cases or not at all?  b) For cases of diarrhea, how often do you give Zinc; most or all the time, sometimes, but less than half of cases or not at all?  c) For cases of diarrhea, how often do you give Antibiotics; most or all the time, sometimes, but less than half of cases or not at all? | **Medicine** | **Most/all the time** | **Some times** | **Not at all** |  |
|  |  | a) ORS | 1 | 2 | 3 |  |
|  |  | b) Zinc | 1 | 2 | 3 |  |
|  |  | c) Antibiotics | 1 | 2 | 3 |  |
| 402 | What antibiotics do you most often prescribe for treating non-bloody diarrhea? | Do not provide antibiotics 1  Metronidazole or combination  product including metronidazole 2  Amoxycillin 3  Cotrim 4  Amox-clavulanate 5  Cefixime 6  Cefotaxime 7  Cefpodoxime 8  Other (Specify)__________________ 96 | | | |  |
| 403 | What antibiotic do you most often prescribe specifically for treating bloody diarrhea? | Do not provide antibiotics 1  Metronidazole 2  Cotrim 3  Cefixime 4  Cefotaxime………………………….5  Cefpodoxime 6  Other (specify)___________________ 96 | | | |  |
| 404 | For respiratory infections, how do you determine which cases should be treated with antibiotics?  ***(Circle all mentioned)*** | Treat all A  Based on respiratory rate B  Fever C  General condition D  Physical examination—including use  of stethoscope listening to the  chest, chest in-drawing E  Other (Specify)___________________X | | | |  |
| 405 | What antibiotic do you most often prescribe for treatment of respiratory infection or pneumonia in infants or young children? | Amoxycillin 1  Cotrim 2  Amox-clavulanate 3  Cefixime 4  Cefotaxime 5  Cefpodoxime 6  Azithromycin 7  Other (Specify)__________________ 96 | | | |  |
| 406 | What is the most common formulation of antibiotics used? | Oral tablets/capsules 1  Dispersible tablets 2  Syrup/ suspension 3  Injections 4  Others (Specify)_______________ 96 | | | |  |
| **Now I would like to talk to you about sick young infants 0-2 months of age** | | | | | | |
| 407 | Are treatments given from this clinic for illnesses among young infants 0-2 months of age? | Yes, for some conditions 1  No, not at all 2 | | | | End |
| 408 | Over the past **6** months, have any sick young infants 0-2 months been treated using antibiotics (oral or injectable) from this clinic? | Yes 1  No 2 | | | | End |

| **Date of Interview:** | **Day Month Year** |
| --- | --- |

**Section A: Background Characteristics**

Now, I would like to ask you some general information about you.

| **S.No.** | | **Questions** | | | | | | **Responses** | | | | | | | | | | | | | | **Go To** | |
| --- | --- | --- | --- | --- | --- | --- | --- | --- | --- | --- | --- | --- | --- | --- | --- | --- | --- | --- | --- | --- | --- | --- | --- |
| 500 | | **Background Characteristics** | | | | | |  | | | | | | | | | | | | | |  | |
| 501 | | Sex | | | | | | Male 1  Female 2 | | | | | | | | | | | | | |  | |
| 502 | | How old are you? | | | | | | Completed years  | | | | | | | | | | | | | |  | |
| 503 | | What is your highest academic qualification, related to medical care? | | | | | | MBBS doctor 1  Pediatrician…………………………….2  General Practitioner……………………3  Physician……………………………....4  Others (Specify)_________________ 96 | | | | | | | | | | | | | |  | |
| 504 | | Have you ever received training on IMCI/IMNCI? | | | | | | Yes 1  No 2 | | | | | | | | | | | | | |  | |
| 505 | | How long have you been in private practice treating sick young infants (at this clinic or another place)?  ***(Write in months***  ***If <1 month, write “000”)*** | | | | | | Months……………………… | | | | | | | | | | | | | |  | |
| 506 | | How long have you been working in this clinic?  ***(Write in months***  ***If <1 month, write “000”)*** | | | | | | Months……………………… | | | | | | | | | | | | | |  | |
| 507 | | What are the hours this clinic is usually open?  ***(use 24 hh clock; hh:mm)*** | | | | | | a) Open for 24 hours……………………….0  b) Not open for 24 hours…………………1  **Hour Minute**  Opening time:  :   Closing time  :  | | | | | | | | | | | | | |  | |
| 508 | | How many days per week is this clinic open? | | | | | | Days  | | | | | | | | | | | | | |  | |
| 509 | | How many hours per day are you (or another health worker) usually available to see patients in this clinic? | | | | | | Hours  | | | | | | | | | | | | | |  | |
| 510 | | Where do you work besides here?  ***(Circle all mentioned)*** | | | | | | Government hospital A  Private hospital ...B  Other government sector health facility..C  Other private health sector, health Facility D  Private drug shop E  Other private sector, non-healthcare F  Nowhere else Y | | | | | | | | | | | | | |  | |
| 511 | | Does this clinic provide inpatient care for sick young infants 0-2 months? | | | | | | Yes 1  No 2 | | | | | | | | | | | | | |  | |
| 512 | | Does this clinic use one specific hospital for referral of infants, with serious illness, needing higher level care? | | | | | | Yes 1  No 2 | | | | | | | | | | | | | | 514 | |
| 513 | | For such referral cases, what specific hospital do you usually refer them to?  ***a) Record name and probe to determine:***  ***b) whether facility is public or private?***  ***c) within the district or beyond?***  ***d) time to travel there by usual transport?*** | | | | | | **a) Name of hospital:** _____________  **b)** **Type**  - Government hospital 1  - Private hospital 2  -Others (Specify)______________96  **c)** **Location**  - Within district 1  - Beyond district 2  **d)** **Time by usual transport**  - <30 minutes 1  - 30 <1 hours 2  - 1+ hours 3 | | | | | | | | | | | | | |  | |
| 514 | | Do you provide inpatient care (treatment) for sick young infants 0-2 months old, admitted either in this clinic or elsewhere?  ***(If yes, then probe: Where do you provide inpatient care for such cases?)*** | | | | | | No role in providing inpatient care 1  Provide inpatient care for infants at  associated hospital 2  Provide inpatient care for sick  young infants at this clinic 3  Other (Specify)__________________ 96 | | | | | | | | | | | | | |  | |
| **Section B: Assessment, treatment and referral of sick young infants 0-2 months**  Now I would like to ask you some questions regarding the assessment,treatment and referral practices of sick young infants 0-2 months of age, who come to this clinic. | | | | | | | | | | | | | | | | | | | | | | | |
| ***Assessment*** | | | | | | | | | | | | | | | | | | | | | | | |
| 515 | | What actions do you normally take to assess sick young infants 0-2 months at this clinic?  ***(Circle all mentioned)*** | | | | | | Take temperature A  Count respiratory rate B  Check oxygen saturation with pulse  oximeter C  Listen to the patient’s breathing using  stethoscope D  Weigh child E  Ask caregiver about how infant is  feeding F  Ask caregiver about whether or not  they have observed the baby  convulsing G  Assess child’s movement/level of  consciousness H  Assess chest indrawing………………….I  Assess if bulging fontanelle present…….J  Determine age of the infant K  Listen for grunting L  Check for skin pustules M  Check for redness/pus in umbilical region ….N  Jaundice ….O  Other (Specify)____________________X  No action taken Y | | | | | | | | | | | |  | | | |
| 516 | | What specific equipment/instruments do you use to assess sick young infants 0-2 months?  ***(Circle all mentioned)*** | | | | | | Respiratory rate timer/watch A  Thermometer B  Stethoscope C  Pulse oximeter D  Tongue depressor E  Other (Specify)____________________X  No equipment used Y | | | | | | | | | | | |  | | | |
| 517 | | What, if any, reference materials/job-aids do you use for assessment/ classification (to see how sick they are) of sick young infants?  ***(Circle all mentioned)*** | | | | | | IMNCI treatment guideline A  CIMS B  MIMS C  Course books D  Other (Specify)____________________ X  No reference materials used Y | | | | | | | | | | | |  | | | |
| 518 | | For each of the following signs of illness in sick young infants 0-2 months, indicate, if present, whether you would normally: treat out-patient, treat inpatient, refer to hospital or are unable to assess: | | | | | | | | | **Treat outpatient** | **Treat inpatient** | **Refer to hospital** | **Unable to assess** | | | | | |  | | | |
| 1 | | Unconscious or abnormally reduced level of consciousness | | | | | | | | | 1 | 2 | 3 | 4 | | | | | |  | | | |
| 2 | | Convulsion/history of convulsion | | | | | | | | | 1 | 2 | 3 | 4 | | | | | |  | | | |
| 3 | | Persistent vomiting | | | | | | | | | 1 | 2 | 3 | 4 | | | | | |  | | | |
| 4 | | Central cyanosis | | | | | | | | | 1 | 2 | 3 | 4 | | | | | |  | | | |
| 5 | | Unable to feed | | | | | | | | | 1 | 2 | 3 | 4 | | | | | |  | | | |
| 6 | | Bulging fontanelle | | | | | | | | | 1 | 2 | 3 | 4 | | | | | |  | | | |
| 7 | | Weight <1,500g | | | | | | | | | 1 | 2 | 3 | 4 | | | | | |  | | | |
| 8 | | Weight < another threshold (Specify)________ | | | | | | | | | 1 | 2 | 3 | 4 | | | | | |  | | | |
| 9 | | Blood oxygen saturation (SpO2) <90% or another threshold (Specify)________________ | | | | | | | | | 1 | 2 | 3 | 4 | | | | | |  | | | |
| 10 | | Fast breathing | | | | | | | | | 1 | 2 | 3 | 4 | | | | | |  | | | |
| 11 | | Fever | | | | | | | | | 1 | 2 | 3 | 4 | | | | | |  | | | |
| 12 | | Abnormally low temperature (<35.5^0^ C) | | | | | | | | | 1 | 2 | 3 | 4 | | | | | |  | | | |
| 13 | | Movement only with stimulation | | | | | | | | | 1 | 2 | 3 | 4 | | | | | |  | | | |
| 14 | | Severe chest-in drawing | | | | | | | | | 1 | 2 | 3 | 4 | | | | | |  | | | |
| 15 | | Poor feeding | | | | | | | | | 1 | 2 | 3 | 4 | | | | | |  | | | |
| ***Treatment*** | | | | | | | | | | | | | | | | | | | | | | | |
| 519 | | About how many sick infants (0-2 months of age) have you treated over the past 6 months from this clinic?  ***(Specify for outpatient and inpatient)***  ***(If > 995, write 995)*** | | | | | | a) Estimated No. of cases ≤2 months  treated as outpatient   b) Estimate No. of cases ≤2 months  treated as inpatient   c) Don’t know…………………………98 | | | | | | | | | | | |  | | | |
| 520 | | About how many of these were 0-1 month of age?  ***(Specify for outpatient and inpatient)***    ***(If > 995, write 995)*** | | | | | | a) Estimated No. of cases ≤1 months  treated as outpatient   b) Estimate No. of cases ≤1 months  treated as inpatient   c) Don’t know…………………………98``` | | | | | | | | | | | |  | | | |
| 521 | | | What types of treatments do you provide to sick young infants 0-2 months?  ***(Prompt ‘anything else?’ Circle all mentioned)*** | | | | | | | Oral antibiotics A  Injectable antibiotics B  Bronchodilators C  Steroids D  Other (Specify)_________________ X | | | | | | | | | | |  | | |
| 522 | | About how many of them (0-2 months olds) did you prescribe oral antibiotics for treatment? | | | | | | None of them 1  Some of them (i.e. less than half) 2  Most of them (i.e. more than half) 3  All of them 4  Don’t know 98 | | | | | | | | | | | | 528 | | | |
| 523 | | What signs do you use to determine which sick young infants 0-2 months need oral antibiotics?  ***(Circle all mentioned)*** | | | | | | Treat all A  Based on respiratory rate/  fast-breathing B  Fever C  Physical examination—including use  of stethoscope listing to the chest D  General condition – looks unwell E  Severe chest indrawing F  Other (Specify)___________________ X  Don’t know Z | | | | | | | | | | | |  | | | |
| 524 | | What specific oral antibiotic do you normally prescribe as1^st^ line for treating sick young infants 0-2 months old in this clinic?  ***(Circle all mentioned)*** | | | | | | | Amoxycillin A  Cotrim B  Amox-clavulanate C  Azithromycin D  Cefixime E  Cefotaxime F  Cefpodoxime G  Other (Specify)___________________ X  Don’t know Z | | | | | | | | | | |  | | | |
| 525 | | Which formulation(s) of oral antibiotic do you normally prescribe for young infants?  ***(Circle all mentioned)*** | | | | | | | Oral suspension/ syrup A  Dispersible tablets B  Non-dispersible tablets/capsules C  Drops D  Other (Specify)___________________ X  Don’t know Z | | | | | | | | | | |  | | | |
| 526 | | For the specific oral antibiotics used as 1^st^ line, kindly give name, usual dosage used (by weight or weight band), frequency* (OD, BID, TDS,QID), duration (# of days):  (* OD=1, BID=2, TDS=3 and QID=4)  ***Note: Write the names of medicines selected in Ques. 524, in the column below***   \| S.N \| Drug Name \| Strength / concentration of medicine \| Formulation: \| Dosage \| \| Frequency per day* \| Duration  (# of days) \| \| \| --- \| --- \| --- \| --- \| --- \| --- \| --- \| --- \| --- \| \| Syrup/ suspension=1  Dispersible tablet=2  Non-dispersible tablet/ capsule = 3  Drop = 4  Others (Specify) =96 \| Unit  Mg/ Kg=1  Ml=2  Mg=3  Gm=4  Others (Specify)=96 \| Amount \| \| 1 \| Amoxycillin \|  \|  \|  \|  \|  \| 1 \| \| \| 2 \| Cotrim \|  \|  \|  \|  \|  \|  \| \| \| 3 \| Amox-clavulanate \|  \|  \|  \|  \|  \|  \| \| \| 4 \| Azithromycin \|  \|  \|  \|  \|  \|  \| \| \| 5 \| Cefixime \|  \|  \|  \|  \|  \|  \| \| \| 6 \| Cefotaxime \|  \|  \|  \|  \|  \|  \| \| \| 7 \| Cefpodoxime \|  \|  \|  \|  \|  \|  \| \| \| 8 \| Others 1____ \|  \|  \|  \|  \|  \|  \| \| \| 9 \| Others 2____ \|  \|  \|  \|  \|  \|  \| \| \| 10 \| Others 3____ \|  \|  \|  \|  \|  \|  \| | | | | | | | | | | | | | | | | | |  | | | |
| 527 | | For infants for whom you have prescribed oral antibiotics – do you usually administer the first dose to them at the clinic (or adjoining medical shop)? | | Yes, for all cases 1  Yes, in some cases 2  No 3 | | | | | | | | | | | | | | | |  | | | |
| 528 | | Over the past 6 months, about how many sick young infants 0- 2months old did you treat using injectable antibiotics, in this clinic?  ***(If > 995, write 995)*** | | No. of ≤ 2 months treated with injectable  antibiotics   None…………………………………...0  Don’t know 998 | | | | | | | | | | | | | | | | 535 | | | |
| 529 | | About how many of these were 0-1 month of age?  ***(If > 995, write 995)*** | | No. of ≤1 months treated with injectable  antibiotics   None…………………………….………..0  Don’t know 998 | | | | | | | | | | | | | | | |  | | | |
| 530 | | What signs do (did) you use to determine which young infants 0-2 months need injectable antibiotics?  ***(Circle all mentioned)*** | | Treat all A  Based on respiratory rate  (fast breathing) B  Fever C  Low temperature D  Severe chest-indrawing E  Poor feeding F  Movement only with stimulation G  Convulsion…………………………….H  Other (Specify)___________________X | | | | | | | | | | | | | | | |  | | | |
| 531 | | What injectable antibiotic(s) do (did) you normally use as 1^st^ line for treating sick young infants 0-2months old?  ***(Probe “any others?” and circle all mentioned)*** | | Gentamycin A  Ampicillin B  Penicillin C  Ceftriaxone D  Cefuroxime E  Cefotaxime F  Other (Specify)___________________X  Don’t know Z | | | | | | | | | | | | | | | |  | | | |
| 532 | | For the specific injectable antibiotics used as 1^st^ line, Kindly give usual dosage used (by weight or weight band), frequency (OD, BID, TDS,QID), duration (# of days; OD=1, BID =2, TDS=3 and QID=4)*  ***Note: Write the name of medicines selected in Ques. 531, in the column below***   \| S.N \| Drug Name \| Strength / concentration of medicine \| Dosage \| \| Frequency per day* \| Duration  (# of days) \| \| --- \| --- \| --- \| --- \| --- \| --- \| --- \| \| Amount \| Unit  Mg/ Kg=1  Ml=2  Mg=3  Gm=4  Others (specify)=96 \| \| 1 \| Gentamycin \|  \|  \|  \|  \|  \| \| 2 \| Ampicillin \|  \|  \|  \|  \|  \| \| 3 \| Penicillin \|  \|  \|  \|  \|  \| \| 4 \| Ceftriaxone \|  \|  \|  \|  \|  \| \| 5 \| Cefuroxime \|  \|  \|  \|  \|  \| \| 6 \| Cefotaxime \|  \|  \|  \|  \|  \| \| 7 \| Others 1____ \|  \|  \|  \|  \|  \| \| 8 \| Others 2____ \|  \|  \|  \|  \|  \| \| 9 \| Others 3____ \|  \|  \|  \|  \|  \| | | | | | | | | | | | | | | | | | |  | | | |
| **S.No.** | **Questions** | | | | | | **Responses** | | | | | | | | | | | | | **Go To** | | | |
| 533 | What injectable antibiotics do you normally use as 2^nd^ line for treating sick young infants 0-2months old (i.e. for cases that have not adequately responded to earlier antibiotic treatment)?  ***(Circle all mentioned)*** | | | | | | Gentamycin A  Ampicillin B  Penicillin C  Ceftriaxone D  Cefuroxime E  Cefotaxime F  Other (Specify)___________________X  Never use / Refer Y  Don’t know Z | | | | | | | | | | | | | 535 | | | |
| 534 | For the 2^nd^ line injectable antibiotics used, kindly give usual dosage normally used (by weight or weight band), frequency (OD, BID, TDS,QID), duration (# of days): Write ( OD=1, BID =2, TDS=3 and QID=4)*  ***Note: Write the name of medicines selected in Ques. 533, in the column below***   \| S.N \| Drug Name \| Strength / concentration of medicine \| Dosage \| \| Frequency per day* \| Duration  (# of days) \| \| --- \| --- \| --- \| --- \| --- \| --- \| --- \| \| Amount \| Unit  Mg/ Kg=1  Ml=2  Mg=3  Gm=4  Others (specify)=96 \| \| 1 \| Gentamycin \|  \|  \|  \|  \|  \| \| 2 \| Ampicillin \|  \|  \|  \|  \|  \| \| 3 \| Penicillin \|  \|  \|  \|  \|  \| \| 4 \| Ceftriaxone \|  \|  \|  \|  \|  \| \| 5 \| Cefuroxime \|  \|  \|  \|  \|  \| \| 6 \| Cefotaxime \|  \|  \|  \|  \|  \| \| 7 \| Others 1____ \|  \|  \|  \|  \|  \| \| 8 \| Others 2____ \|  \|  \|  \|  \|  \| \| 9 \| Others 3____ \|  \|  \|  \|  \|  \| | | | | | | | | | | | | | | | | | | |  | | | |
| 535 | What reference materials/job-aids, if any, do you use to determine appropriate antibiotics and doses for treating sick young infants?  ***(Circle all mentioned)*** | | | | | IMNCI treatment guideline A  CIMS B  MIMS C  Course books D  Others X  No reference materials used Y | | | | | | | | | | | | |  | | | |  |
| 536 | How do you determine the appropriate antibiotic dose? | | | | | By age 1  By weight 2  Other (Specify)__________________ 96 | | | | | | | | | | | | | 540  540 | | | |  |
| 537 | Do you determine dose: by mg/kg or by weight bands? | | | | | By mg/kg 1  Weight bands 2  Other (Specify)__________________ 96 | | | | | | | | | | | | |  | | | |  |
| 538 | How do you determine the baby’s weight?  ***(Probe to determine which type of scale is used if respondent indicates they weigh the child)*** | | | | | Weigh baby using adult scale  (difference method) 1  Weigh baby using Salter scale 2  Weigh baby using Pan scale 3  Estimate weight by looking at child 4 | | | | | | | | | | | | | 540 | | | |  |
| 539 | Is weight determined leaving the baby’s coverings on or removing them? | | | | | Leaving baby’s clothes on 1  Removing baby’s clothes 2 | | | | | | | | | | | | |  | | | |  |
| 540 | How many days of injectable treatment do you normally give to sick young infants 0-2 months as a minimum? | | | | | No. of days  | | | | | | | | | | | | |  | | | |  |
| 541 | How common it is for shorter treatment to be given due to failure to return to the clinic, or parents’ inability to pay for more treatment. | | | | | Very common (i.e. more than half  the time) 1  Somewhat common 2  Not very common 3  Never happens 4 | | | | | | | | | | | | |  | | | |  |
| 542 | For cases that you don’t refer on to hospital, how often is it that only a single dose of injection is given (i.e. rather than have the baby brought back for another dose the next day)? | | | | | Very common (i.e. more than half  the time) 1  Somewhat common 2  Not very common 3  Never happens 4 | | | | | | | | | | | | |  | | | |  |
| 543 | How common is it for you to administer a single injection and then refer the baby to the hospital? | | | | | Very common (i.e. more than half  the time) 1  Somewhat common 2  Not very common 3  Never happens 4 | | | | | | | | | | | | |  | | | |  |
| 544 | Under what circumstances, if any, do you prescribe injectable steroids (e.g. dexamethasone) for treating sick young infants 0-2 months?  ***(Circle all mentioned)*** | | | | | When child has signs of critical  illness A  When child is not responding to  initial treatment B  Other (Specify)___________________ X  Never Y | | | | | | | | | | | | | 547 | | | |  |
| 545 | How common is it for you to administer injectable steroids to sick young infants 0-2 months? | | | | | Very common 1  Somewhat common 2  Not very common 3  Never happens 4 | | | | | | | | | | | | |  | | | |  |
| 546 | Over the past 6 months, about how many sick young infants 0-2months old did you treat using injectable steroids (like dexamethasone)?  ***(If > 995, write 995)*** | | | | | None 0  No. treated with steroids   Don’t know 998 | | | | | | | | | | | | |  | | | |  |
| ***Referral*** | | | | | | | | | | | | | | | | | | | | | | |  |
| 547 | | What are the signs that indicate a child has severe illness and requires referral to hospital?  ***(Circle all mentioned)*** | | | | Unconscious or drowsy A  Convulsion/history of convulsion B  Persistent vomiting C  Central cyanosis (appears blue) D  Unable to feed E  Bulging fontanelle F  Too small/weight <1,500g G  Continuing illness despite treatment H  Age cut-off I  Other (Specify)___________________ X  Don’t know Z | | | | | | | | | |  | | | | | | |  |
| ***Now I would like to ask you some questions about your referral practices for young infants with very severe illness (e.g. Unconscious or drowsy; convulsion/history of convulsion; persistent vomiting; central cyanosis; unable to feed; bulging fontanelle; weight <1,500g)*** | | | | | | | | | | | | | | | | | | | | | | |  |
| 549 | | For cases that you refer to higher level care do you give any treatment before referral? If yes, then what treatment, do you give before referral?  ***(Circle all mentioned)*** | | | | No pre-referral treatment A  Oral Antibiotics B  Injectable Antibiotics C  Other (Specify)___________________X | | | | | | | | | | | | 551  551  551 | | | | |  |
| 550 | | For pre-referral injectable antibiotics, what specific drug(s) do you normally give as first line? | | | Gentamycin 1  Ampicillin 2  Penicillin 3  Ceftriaxone 4  Cefuroxime 5  Cefotaxime 6  Other (Specify)___________________96 | | | | | | | | | | | |  | | | | | |  |
| 550.1 | | What dose of that antibiotic do you give (specific dose)? | | | 1. Mg / kg…………... **.**   2. Mg……………….. **.**   3. Ml………………... **.**   4. Gm……………….. **.**   5. Others (Specify) ______________996 | | | | | | | | | | | |  | | | | | |  |
| 551 | | Beyond telling the parents where to take the baby, do you normally do anything else to facilitate referral?  ***(Circle all mentioned)*** | | | Help arrange transport/ambulance A  Provide referral note/slip B  Call ahead/communicate with  physician or other staff at receiving  institution C  Counsel on importance of  completing referral immediately D  Other (Specify) ___________________X | | | | | | | | | | | |  | | | | | |  |
| 552 | | How often have young infants, you have referred out for treatment, returned to you after being discharged for completion of treatment or without having been to the referred out center? | | | Very common (i.e. more than half) 1  Somewhat common 2  Not very common 3  Never happens 4 | | | | | | | | | | | |  | | | | | |  |
| ***Section C: Follow-up and counseling of sick young infants given antibiotics(Oral or injectable)*** | | | | | | | | | | | | | | | | | | | | | | |  |
| *Now I would like to ask you some questions about counseling and follow-up of sick young infants 0-2 months for whom you have begun treatment with antibiotics* | | | | | | | | | | | | | | | | | | | | | | |  |
| 553 | | For non-referred cases, what is the minimum schedule of further contacts, if any, you normally have with these patients (beyond the initial contact)? | | | Daily contact 1  Specific days 2  No further contacts made 3 | | | | | | | | | | 553.1  553.1  554 | | | | | | | |  |
| 553.1 | | If **daily contact**, then for how many days? And  If follow- up on **specific days**, then write the days.  ***(Probe upto 3 times to determine what specific days the child is re-assessed)*** | | | Daily contact ……………………………1  Total no. of Days of contact______  Specific days………………………2  Day _____ Day ____ Day ____ | | | | | | | | | |  | | | | | | | |  |
| 554 | | For non-referred cases, on what day(s) do you reassess the child and make a decision about whether to continue treatment or refer?  ***(Circle all mentioned)*** | | | Day 2 of treatment A  Day 3 of treatment B  Day 4 of treatment C  After Day 4 of treatment D  Other (Specify)___________________X | | | | | | | | | |  | | | | | | | |  |
| 555 | | What advice do you normally give parents/ guardians at the time treatment is started?  ***(Circle all mentioned)*** | | | Instructions on administering oral  antibiotics A  Danger signs to look for and where  they should go B  When they should bring the baby  back for the next follow-up/for  next injection C  Other (Specify)___________________ X  No advice given Y | | | | | | | | | |  | | | | | | | |  |
| 556 | | What do you do if the caregiver cannot afford the full course of treatment?  ***(Circle all mentioned)*** | | | Shorten the course of treatment A  Offer deferred payment option B  Suggest treatment with lower cost  medicines C  Refer to public facility D  Other (Specify)___________________ X  Don’t know/never happens Z | | | | | | | | | |  | | | | | | | |  |
| 557 | | What do you do if the infant develops side effects to treatment?  ***(Circle all mentioned)*** | | | Discontinue treatment A  Refer to hospital B  Switch to alternate medicine C  Other (Specify)___________________ X  Don’t know/never happens Z | | | | | | | | | |  | | | | | | | |  |
| 558 | | What do you do if caregivers refuse to continue treatment?  ***(Circle all mentioned)*** | | | Counsel on importance of completing  treatment A  Refer B  Offer an alternate treatment C  Discontinue treatment D  Other (Specify)___________________ X  Don’t know/never happens Z | | | | | | | | | |  | | | | | | | |  |
| 559 | | Under what circumstances, if any, do you decide to shorten the course of antibiotic treatment in a sick young infant?  ***(Circle all mentioned)*** | | | When child’s condition improves A  If caregivers cannot afford full course B  If child develops side effects C  If caregivers refuse to continue D  Other (Specify)___________________ X  Never abbreviate course Y | | | | | | | | | |  | | | | | | | |  |
| 560 | | What actions, if any, are taken for those who do not return for follow-up as expected?  ***(Circle all mentioned)*** | | | Phone family A  Send someone to find the family B  Other (Specify)___________________ X  No action is taken Y | | | | | | | | | |  | | | | | | | |  |

| **Section D. Observation of equipment and reference materials for management of sick young infants**  ***During assessment and treatment of sick young infants, the service provider may have used the following equipment/ instruments and reference materials. Observe those, and in case they are not easily seen, ask the service provider, observe and then select ONE appropriate code for each item.*** | | | | |
| --- | --- | --- | --- | --- |
| **561. Determine availability and functionality for each item of equipment listed below** | | **Available and functioning** | **Available but not useable** | **Not available** |
|  | Salter scale | 1 | 2 | 3 |
|  | Pan scale | 1 | 2 | 3 |
|  | Other infant scale (Specify)____________________ | 1 | 2 | 3 |
|  | Adult scale | 1 | 2 | 3 |
|  | Thermometer (digital) | 1 | 2 | 3 |
|  | Thermometer (other) | 1 | 2 | 3 |
|  | Stethoscope | 1 | 2 | 3 |
|  | Pulse oximeter | 1 | 2 | 3 |
|  | Timer/watch for counting respiratory rate | 1 | 2 | 3 |
|  | Cell phone | 1 | 2 | 3 |
| **561. Ask to see the following reference materials/job aids and other support materials.** | | **Available** | **Not available** |  |
|  | IMNCI treatment guideline | 1 | 2 |  |
|  | CIMS | 1 | 2 |  |
|  | MIMS | 1 | 2 |  |
|  | Other relevant reference materials (Specify)_________ |  |  |  |
|  | Register for recording sick child cases | 1 | 2 |  |
