# Supplementary material for: A national survey of private-sector outpatient care of sick infants and young children in Nepal
Source: BMC Health Serv Res. 2020 Jun 16;20:545. doi: 10.1186/s12913-020-05393-1 (PMC7298835; doi:10.1186/s12913-020-05393-1)
Supplement: Supplementary file 1 — Additional file 1. [file 12913_2020_5393_MOESM1_ESM.zip › Medical shop_survey instrumentsR4.docx]

**A National Survey on Care of Possible Serious Bacterial Infection (PSBI) among Sick Young Infants 0-2 months in Private Sector Medical Shops and Clinics in Nepal**

**(Medical Shops)**

**Screening questionnaire to identify Medical Shops that treat sick young infants 0-2 months with antibiotics**

***Note: Ask to speak to the main person responsible for treating sick young infants. If they are not available on first attempt, schedule a time to return when they will be able to complete the interview.***

**General Information**

| **District Code** | |  |
| --- | --- | --- |
| **VDC/Municipality Name and Code**: ________________ | |  |
| **Name of new administrative structure (Palika)** | | ………………………. |
| **Ward No.** | |  |
| **Name of Medical Shop:** ________________________________  **Questionnaire No**…**………………………………………………………………** | | |
| **Type of Strata (refer to the sampling)** | Proximal (half an hour distance from hospital) 1  Semi Proximal (30 minutes to 1 hour) 2  Remote (more than 1 hours) 3 | |
| **Where is this medical shop registered?**  ***(More than one response possible; circle all that apply)*** | Sangini registered A  DDA registered B  NCDA registered C  No registration D | |
| **Interviewer code………………….……..** |  | |
| **Date of Screening (A.D)** |     Day Month Year | |
| **GPS (Latitude)……..………………….…** |  .  | |
| **GPS (Longitude)………………….………** |  .  | |
| **Survey type** | National  Pilot  | |

**Questions about management of diarrhea and respiratory infections among young children aged 2-59 months**

| ***Administer Consent Form***  **Consent Given…………………… Start the interview **  **Consent Not Given……………… Thank the Respondent and END the interview ** |
| --- |

**Now I would like to ask you some questions about Management of Diarrhea and Respiratory Infections among young child 2-59 months**

| **S.No.** | **Questions** | **Responses** | | | | **Go To** |
| --- | --- | --- | --- | --- | --- | --- |
| 100 | For children with diarrhea, what decisions do you make at this shop?  ***(Note: Read response options and circle most appropriate response)*** | Dispensing drugs 1  Treatment services 2  Both dispense and treat 3  Referral only 4 | | | | 104  104 |
| 101 | a) For cases of diarrhea, how often do you give ORS; most or all the time, sometimes, but less than half of cases or not at all?  b) For cases of diarrhea, how often do you give Zinc; most or all the time, sometimes, but less than half of cases or not at all?  c) For cases of diarrhea, how often do you give Antibiotics; most or all the time, sometimes, but less than half of cases or not at all? | **Medicine** | **Most/all the time** | **Some times** | **Not at all** |  |
|  |  | a) ORS | 1 | 2 | 3 |  |
|  |  | b) Zinc | 1 | 2 | 3 |  |
|  |  | c) Antibiotics | 1 | 2 | 3 |  |
| 102 | What antibiotic do you most often give for treating non-bloody diarrhea? | Do not provide antibiotics 1  Metronidazole or a combination  antibiotic product that includes  metronidazole 2  Amoxycillin 3  Cotrim 4  Amox-clavulanate 5  Cefixime 6  Cefotaxime 7  Cefpodoxime 8  Other (Specify)_______________96 | | | |  |
| 103 | What antibiotic do you most often give specifically for treating bloody diarrhea? | Do not provide antibiotics 1  Metronidazole or a combination antibiotic product that includes metronidazole……………………..2  Cotrim 3  Cefixime 4  Cefotaxime…………………………5  Cefpodoxime 6  Other (Specify)_______________96 | | | |  |
| 104 | For children with respiratory infection, what do you do at this shop; only dispense drugs / treat / dispense and treat both or only refer?  ***(Note: Read response options and circle most appropriate response)*** | Dispensing drugs 1  Treatment services 2  Both dispense and treat 3  Referral only 4 | | | | 108  108 |
| 105 | For respiratory infections, how do you determine which cases should be treated with antibiotics?  ***(Circle all mentioned)*** | Treat all A  Based on respiratory rate B  Fever C  General Condition D  Physical examination—including use of stethoscope listening to the chest, chest in-drawing E  Other (Specify)________________X | | | |  |
| 106 | What antibiotic do you most often give for treating respiratory infections or pneumonia in infants or young children ? | Amoxycillin 1  Cotrim 2  Amox-clavulanate 3  Cefixime 4  Cefotaxime…………………………5  Cefpodoxime 6  Azithromycin 7  Other (Specify)_______________ 96 | | | |  |
| 107 | What is the most common formulation of antibiotics used? | Oral tablets/ capsules 1  Dispersible tablets 2  Syrup/ suspension 3  Injections 4  Others (Specify)_______________ 96 | | | |  |

| **S.No.** | **Questions** | **Responses** | **Go To** |
| --- | --- | --- | --- |
| **Screening questions for PSBI care:** | | | |
| 108 | What is the availability of a physician at this outlet? | Physician available throughout  the week 1  Physician available for a certain number of days of a week 2  Physician available for a certain number of days in a month 3  Physician not available at this shop 4 | Enter  07 in 108.1    109 |
| 108.1 | Number of days of availability of a physician at this outlet? | Days a week 1  Days a month 2 |  |
| 108.2 | Number of hours of availability of a physician at this outlet, in a day? | Hours in day  |  |
|  | **Now I would like to talk to you about sick young infants 0-2 months of age** | |  |
| 109 | Are treatments given or medicine dispensed from here for illnesses among young infants 0-2 months of age? | Yes, for some conditions 1  No, not at all 2 | END |
| 110 | Over the past **6** months, have any sick young infants 0-2months been treated with antibiotics (oral or injectable) at this drug shop? | Yes 1  No 2 | Tool 1 |
| 110.A | Over the past **6** months, have any sick young infants 0-2months been treated with other medicines (other than antibioitics) or steroids at this drug shop? | Yes 1  No 2 |  |
| 111 | Over the past **6** months, have any sick young infants 0-2months been been distributed antibiotics from this medicine shop? | Yes 1  No 2 | tool 2  End interview |

**Interviewer:**

- **End the interview if the drug shop has not treated any sick young infants 0-2 months with antibiotics in the last 6 months**
- **Administer Tool #1 to drug shops involved in assessment and decision-making around treatment of sick young infants.**
- **Administer Tool #2 to drug shops that only dispense treatments and are not involved in decision-making.**

| **Date of Interview:** | **Day Month Year** |
| --- | --- |

**Tool 1. Providers at private drug shops who assess and treat sick young infants 0-2 months**

**200.** **Section A: Background Characteristics**

Now I would like to ask you some general information about you.

| **S.No.** | **Questions** | **Responses** | **Go To** |
| --- | --- | --- | --- |
| 201 | Sex | Male 1  Female 2 |  |
| 202 | How old are you? | Completed years  |  |
| 203 | What is your highest academic qualification, related to medical care? | Medical doctor 1  Nurse 2  ANM 3  Pharmacy assistant/Pharmacist 4  HA 5  CMA 6  No formal Qualification but have  worked as VHW/MCHW 7  No formal Qualification 8  Others (Specify)_______________96 |  |
| 204 | Have you ever received training on IMCI/IMNCI? | Yes 1  No 2 |  |
| 205 | Have you ever received training on CBNCP? | Yes 1  No 2 |  |
| 206 | How long have you been in private practice treating sick young infants (at this shop or another place)?  ***(Write in months***  ***If <1 month, write “000”)*** | Months…………………... |  |
| 207 | How long have you been working in this medicine shop?  ***(Write in months***  ***If <1 month, write “000”)*** | Months…………………... |  |
| 208 | What are the hours this shop is usually open?  ***(use 24 hh clock; hh:mm)*** | a) Open for 24 hours……………………….0  b) Not open for 24 hours…………………1  **Hour Minute**  Opening time: :  Closing time : |  |
| 209 | How many days per week is this shop open? | Days |  |
| 210 | How many hours per day are you (or another health worker) usually available to see patients in this shop? | Hours |  |
| 211 | Where do you work besides here?  ***(Circle all mentioned)*** | Government Health facility A  Other government sector B  Other private health sector facility C  Other private sector, non-healthcare D  Others (Specify)…………………...X  Nowhere else………………………Y |  |
| 212 | Is there usually another qualified health worker (at least CMA qualification) here when you're away? | Yes 1  No 2 |  |

**Section B: Assessment, treatment and referral of sick young infants 0-2 months**

Now I would like to ask you some questions regarding the assessment treatment and referral practices of sick young infants 0-2 months of age, who come to this medical shop.

| ***Assessment*** | | | | | | |
| --- | --- | --- | --- | --- | --- | --- |
| 213 | | What actions do you take to assess sick young infants 0-2 months at this shop?  ***(Circle all mentioned)*** | | Take temperature A  Count respiratory rate B  Check oxygen saturation with pulse  oximeter C  Listen to the patient’s breathing  using stethoscope D  Weigh child E  Ask caregiver about how infant is  feeding F  Ask caregiver about whether or not  they have observed the baby  convulsing G  Assess child’s movement/level of  consciousness H  Assess chest indrawing I  Assess if bulging fontanelle present J  Determine age of the infant K  Listen for grunting L  Check for skin pustules M  Check for redness/pus in umbilical region N  Jaundice O  Other (Specify)________________ X  No action taken Y | |  |
| **S.No.** | | **Questions** | | **Responses** | | **Go To** |
| 214 | | What specific equipment/ instruments do you use to assess sick young infants 0-2 months?  ***(Circle all mentioned)*** | | | Respiratory rate timer/watch A  Thermometer B  Stethoscope C  Pulse oximeter D  Tongue depressor E  Other (Specify)________________ X  No equipment used Y |  |
| 215 | | What, if any, reference materials/job-aids do you use for assessment /classification (to see how sick they are) of sick young infants?  ***(Circle all mentioned)*** | | | IMNCI treatment guideline A  CIMS B  MIMS C  Course books D  Other (Specify) ________________X  No reference materials used Y |  |
| 216 | | What findings from your assessment of sick newborns, 0-2 months age, indicate possible severe or serious illness (PSBI)?  ***(Probe “any others?” and circle all mentioned)*** | | | High fever (>38.5 0C) A  Hypothermia (<35.50C) B  Severe chest in-drawing C  Bulging fontanelle D  Nasal flaring E  Fast breathing F  Convulsion G  No movement or only on stimulation………………………...H  Unconscious I  Poor/no feeding J  Others (Specify)_______________ X  Don’t know Z |  |
| ***Treatment*** | | | | | | |
| 217 | About how many sick infants 0-2 months of age have you treated over the past 6 months at this shop?  ***(If > 995, write 995)*** | | Estimated No. of cases ≤2 months    Don't know 998 | | |  |
| 218 | About how many of these were 0-1 month of age?  ***(If > 995, write 995)*** | | Estimated No. of cases ≤1months    None………………………………0  Don't know 998 | | |  |
| 219 | What types of treatments do you provide to sick young infants 0-2 months?  ***(Read all responses and Prompt ‘anything else?’ Circle all mentioned)*** | | Oral antibiotics A  Injectable antibiotics B  Bronchodilators C  Steroids D  Other (Specify)_________________ X | | |  |
| 220 | About how many of them (0-2 months) did you provide oral antibiotics for treatment? | | None of them 1  Some of them (i.e. less than half) 2  Most of them (i.e. more than half) 3  All of them 4  Don’t know 98 | | | 226 |
| 221 | What signs do you use to determine which sick young infants, 0-2 months, need oral antibiotics?  ***(Circle all mentioned)*** | | Treat all A  Based on respiratory rate/  fast-breathing B  Fever C  Physical examination—including  use of stethoscope listing to the chest D  General condition – looks unwell E  Severe chest indrawing F  Other (Specify)_________________X  Don’t know Z | | |  |
| 222 | What specific oral antibiotic do you normally use as 1^st^ line for treating sick young infants 0-2months old?  ***(Circle all mentioned)*** | | Amoxycillin A  Cotrim B  Amox-clavulanate C  Azithromycin D  Cefixime E  Cefotaxime F  Cefpodoxime G  Other (Specify)________________X  Don’t know ………………………..Z | | |  |
| 223 | Which formulation(s) of oral antibiotic do you normally use for young infants, at this shop?  ***(Circle all mentioned)*** | | Oral suspension/ syrup A  Dispersible tablets B  Non-dispersible tablets/ capsules C  Drops D  Other (Specify)________________X  Don’t know…………………………Z | | |  |
| 224 | For the specific oral antibiotics used as 1^st^ line, please give us the name, usual dosage used (by weight or weight band), frequency* (OD=1, BID=2, TDS=3, QID=4), and duration (# of days):  ***Note: Write the name of medicines selected in Ques. 222, in the column below***   \| S.N \| Drug Name \| Strength / concentration of medicine \| Formulation: \| Dosage \| \| Frequency per day* \| Duration  (# of days) \| \| \| --- \| --- \| --- \| --- \| --- \| --- \| --- \| --- \| --- \| \| Syrup/ suspension=1  Dispersible tablet=2  Non-dispersible tablet/ capsule = 3  Drop = 4  Others (Specify) =96 \| Unit  Mg/ Kg=1  Ml=2  Mg=3  Gm=4  Others (Specify)=96 \| Amount \| \| 1 \| Amoxycillin \|  \|  \|  \|  \|  \| 1 \| \| \| 2 \| Cotrim \|  \|  \|  \|  \|  \|  \| \| \| 3 \| Amox-clavulanate \|  \|  \|  \|  \|  \|  \| \| \| 4 \| Azithromycin \|  \|  \|  \|  \|  \|  \| \| \| 5 \| Cefixime \|  \|  \|  \|  \|  \|  \| \| \| 6 \| Cefotaxime \|  \|  \|  \|  \|  \|  \| \| \| 7 \| Cefpodoxime \|  \|  \|  \|  \|  \|  \| \| \| 8 \| Others 1____ \|  \|  \|  \|  \|  \|  \| \| \| 9 \| Others 2____ \|  \|  \|  \|  \|  \|  \| \| \| 10 \| Others 3____ \|  \|  \|  \|  \|  \|  \| | | | | |  |
| 225 | For young infants for whom you have prescribed oral antibiotics, do you usually administer the first dose to them at the medicine shop? | | Yes, for all cases 1  Yes, in some cases 2  No 3 | | |  |
| 226 | Over the past 6 months, about how many sick young infants 0-2 months old did you treat using injectable antibiotics?  ***(If > 995, write 995)*** | | No. of ≤2 months treated with injectable antibiotics….......  None 0  Don’t know………………………998 | | | 228 |
| 227 | About how many of these were 0-1 month of age?    ***(If > 995, write 995)*** | | No. of ≤1 months treated with injectable antibiotics  None 0  Don’t know………………………998 | | |  |
| 228 | What signs do(did) you normally use to determine which young infants 0-2 months need injectable antibiotics?  ***(Circle all mentioned)*** | | Treat all A  Based on respiratory rate  (fast breathing) B  Fever C  Low temperature D  Severe chest-indrawing E  Poor feeding F  Movement only with stimulation G  Convulsion………………………...H  Other (Specify)________________ X  Have never used injectable antibiotic ..Y | | | 233 |
| 229 | What injectable antibiotic (s) do (did) you normally use as 1^st^ line for treating sick young infants 0-2 months old, at this shop?  ***(Probe “any others?” and circle all mentioned)*** | | Gentamycin A  Ampicillin B  Penicillin C  Ceftriaxone D  Cefuroxime E  Cefotaxime F  Other (Specify)________________ X  Don’t know…………………………Z | | |  |

| 230 | For the specific injectable antibiotics used as 1^st^ line, please give us the name, usual dosage used (by weight or weight band), frequency *(OD=1, BID=2, TDS=3, QID=4), duration (# of days):  ***Note: Write the name of medicines selected in Ques. 229, in the column below***   \| S.N \| Drug Name \| Strength / concentration of medicine \| Dosage \| \| Frequency per day* \| Duration  (# of days) \| \| --- \| --- \| --- \| --- \| --- \| --- \| --- \| \| Amount \| Unit  Mg/ Kg=1  Ml=2  Mg=3  Gm=4  Others (specify)=96 \| \| 1 \| Gentamycin \|  \|  \|  \|  \|  \| \| 2 \| Ampicillin \|  \|  \|  \|  \|  \| \| 3 \| Penicillin \|  \|  \|  \|  \|  \| \| 4 \| Ceftriaxone \|  \|  \|  \|  \|  \| \| 5 \| Cefuroxime \|  \|  \|  \|  \|  \| \| 6 \| Cefotaxime \|  \|  \|  \|  \|  \| \| 7 \| Others 1____ \|  \|  \|  \|  \|  \| \| 8 \| Others 2____ \|  \|  \|  \|  \|  \| \| 9 \| Others 3____ \|  \|  \|  \|  \|  \| | | | | | | |  | |
| --- | --- | --- | --- | --- | --- | --- | --- | --- | --- | --- | --- | --- | --- | --- | --- | --- | --- | --- | --- | --- | --- | --- | --- | --- | --- | --- | --- | --- | --- | --- | --- | --- | --- | --- | --- | --- | --- | --- | --- | --- | --- | --- | --- | --- | --- | --- | --- | --- | --- | --- | --- | --- | --- | --- | --- | --- | --- | --- | --- | --- | --- | --- | --- | --- | --- | --- | --- | --- | --- | --- | --- | --- | --- | --- | --- | --- | --- | --- | --- | --- | --- |
| 231 | What injectable antibiotics do you normally use as 2^nd^ line for treating sick young infants 0-2months old (i.e. for cases that have not adequately responded to earlier antibiotic treatment)?  ***(Circle all mentioned)*** | | | Gentamycin A  Ampicillin B  Penicillin C  Ceftriaxone D  Cefuroxime E  Cefotaxime F  Other (Specify)_________________X  Never use / Refer Y  Don’t know Z | | | | 233 | |
| 232 | For the 2^nd^ line injectable antibiotics used, please give us the name, usual dosage used (by weight or weight band), frequency* (OD=1, BID=2, TDS=3, QID=4), duration (# of days):  ***Note: Write the name of medicines selected in Ques. 231, in the column below***   \| S.N \| Drug Name \| Strength / concentration of medicine \| Dosage \| \| Frequency per day* \| Duration  (# of days) \| \| --- \| --- \| --- \| --- \| --- \| --- \| --- \| \| Amount \| Unit  Mg/ Kg=1  Ml=2  Mg=3  Gm=4  Others (specify)=96 \| \| 1 \| Gentamycin \|  \|  \|  \|  \|  \| \| 2 \| Ampicillin \|  \|  \|  \|  \|  \| \| 3 \| Penicillin \|  \|  \|  \|  \|  \| \| 4 \| Ceftriaxone \|  \|  \|  \|  \|  \| \| 5 \| Cefuroxime \|  \|  \|  \|  \|  \| \| 6 \| Cefotaxime \|  \|  \|  \|  \|  \| \| 7 \| Others 1____ \|  \|  \|  \|  \|  \| \| 8 \| Others 2____ \|  \|  \|  \|  \|  \| \| 9 \| Others 3____ \|  \|  \|  \|  \|  \| | | | | | | |  | |
| 233 | What reference materials/job-aids, if any, do you use to determine appropriate antibiotics and doses for treating sick young infants?  ***(Circle all mentioned)*** | | | | | IMNCI treatment guideline A  CIMS B  MIMS C  Course books D  Others X  No reference materials used Y | |  | |
| 234 | How do you determine the appropriate antibiotic dose? | | | | | By age 1  By weight 2  Other (Specify)________________ 96 | | 238  238 | |
| 235 | Do you determine dose: by mg/kg or by weight bands? | | | | | By mg/kg 1  Weight bands 2  Other (Specify)_______________ 96 | |  | |
| 236 | How do you determine the baby’s weight?  ***(Probe to determine which type of scale is used if respondent indicates they weigh the child)*** | | | | | Weigh baby using adult scale  (difference method) 1  Weigh baby using Salter scale 2  Weigh baby using Pan scale 3  Estimate weight by looking at child 4 | | 238 | |
| 237 | Is weight determined leaving the baby’s coverings on or removing them? | | | | | Leaving baby’s clothes on 1  Removing baby’s clothes 2 | |  | |
| Check Q no 228, if “Y” option was selected or if the respondent has never used injectable antibiotic go to Q240 | | | | | | | | | |
| 238 | How many days of injectable treatment do you normally give to sick young infants 0-2 months, as a minimum? | | | | | No. of days | |  | |
| 239 | How common is it for shorter treatment to be given due to failure to return to the medicine shop, or parents’ inability to pay for more treatment? | | | | | Very common (i.e. more than half  the time) 1  Somewhat common 2  Not very common 3  Never happens 4 | |  | |
| Check Q no 228, if “Y” option was selected or if the respondent has never used injectable antibiotic go to Q240 | | | | | | | | | |
| 240 | For cases that you don’t refer on to hospital, how often is it that only a single dose of injection is given (i.e. rather than have the baby brought back for another dose the next day)? | | | | | | Very common (i.e. more than half  the time) 1  Somewhat common 2  Not very common 3  Never happens 4 |  | |
| 241 | Under what circumstances, if any, do you prescribe injectable steroids (e.g. dexamethasone) for treating sick young infants 0-2 months?  ***(Circle all mentioned)*** | | | | | | When child has signs of critical  illness A  When child is not responding to  initial treatment B  Other (Specify)________________ X  Never Y | 244 | |
| 242 | How common is it for you to administer injectable steroids to sick young infants 0-2 months? | | Very common 1  Somewhat common 2  Not very common 3  Never happens 4 | | | | |  | |
| 243 | Over the past 6 months, about how many sick young infants 0-2 months old did you treat using injectable steroids (like dexamethasone)?  ***(If > 995, write 995)*** | | | | None 0  No. treated with steroids  Don’t know 998 | | |  | |
| ***Referral*** | | | | | | | | | |
| 244 | | What are the signs that indicate a child has severe illness and requires for higher level of care?  ***(Circle all mentioned)*** | | | Unconscious or drowsy A  Convulsion/history of convulsion B  Persistent vomiting C  Central cyanosis (appears blue) D  Unable to feed E  Bulging fontanelle F  Too small/weight <1,500g G  Continuing illness despite treatment H  Age cut-off I  Other (Specify)________________ X | | | |  |
| ***Now I would like to ask you some questions about your referral practices for young infants with severe illness as we have discussed.*** | | | | | | | | | |
| 245 | | For such referral cases, what specific hospital or clinic do you usually refer them to?  ***a)Record name and probe to determine:***  ***b)whether facility is public or private?***  ***c) within the district or beyond?***  ***d) time to travel there by usual transport)?*** | | | **a) Name:_____________________**  **b)Type:**  Government hospital 1  Private hospital 2  Others (Specify) ______________96  **c) Location:**  Within district 1  Beyond district 2  **d)Time by usual transport:**  <30 minutes 1  30-<1 hour 2  1+ hours 3 | | | |  |
| 246 | | For cases that you refer to higher level care do you give any treatment before referral? If yes, then what treatment, do you give before referral?  ***(Circle all mentioned)*** | | | No pre-referral treatment A  Oral Antibiotics B  Injectable Antibiotics C  Other (Specify)________________X | | | | 248  248  248 |
| 247 | | For pre-referral injectable antibiotics, what specific drug(s) do you normally give as first line? | | | Gentamycin 1  Ampicillin 2  Penicillin 3  Ceftriaxone 4  Cefuroxime 5  Cefotaxime 6  Other (Specify)________________96 | | | |  |
| 247.1 | | What dose of that antibiotic do you give (specific dose) | | | 1. Mg / kg…………… **.**  2. Mg………………... **.**  3. Ml………………… **.**  4. Gm………………... **.**  5. Others (Specify) ______________996 | | | |  |

| 248 | Beyond telling the parents where to take the baby, do you normally do anything else to facilitate referral?  ***(Circle all mentioned)*** | Help arrange transport/ambulance A  Provide referral note/slip B  Call ahead/communicate with  physician or other staff at receiving  institution C  Counsel on importance of  completing referral immediately D  Other (Specify) ________________X |  |
| --- | --- | --- | --- |
| 249 | How often have young infants, you have referred out for treatment, returned to you after being discharged for completion of treatment, or without having been to the referred out center? | Very common (i.e. more than half) 1  Somewhat common 2  Not very common 3  Never happens 4 |  |

**Section C: Follow-up and counseling of sick young infants given antibiotics**

| **S.No.** | **Questions** | | **Responses** | **Go To** | |
| --- | --- | --- | --- | --- | --- |
| ***Now I would like to ask you some questions about counseling and follow-up of sick young infants 0-2 months for which you have begun treatment with antibiotics (oral or injectable)*** | | | | | |
| 250 | For non-referred cases, what is the minimum schedule of further contacts you normally have, if any, with these patients (beyond the initial contact)? | Daily contact (specifying # of days) 1  Specific days (specify below) 2  No further contacts made 3 | | | 250.1  250.1  251 |
| 250.1 | If **daily contact**, then for how many days? And  If follow- up on **specific days**, then write the days.  ***(Probe upto 3 times to determine what specific days the child is re-assessed)*** | Daily contact ……………………………1  Total no. of Days of contact _____  Specific days………………………2  Day _____ Day ____ Day ____ | | |  |
| 251 | For non-referred cases, on what day(s) do you reassess the child and make a decision about whether to continue treatment or refer?  ***(Circle all mentioned)*** | Day 2 of treatment A  Day 3 of treatment B  Day 4 of treatment C  After Day 4 of treatment D  Other (Specify)________________X | | |  |
| 252 | What advice do you normally give parents/ guardians at the time treatment is started?  ***(Circle all mentioned)*** | Instructions on administering oral  antibiotics A  Danger signs to look for and where  they should go B  When they should bring the baby  back for the next follow-up/for  next injection C  Other (Specify)________________ X  No advice given Y | | |  |

| 253 | What do you do if the caregiver cannot afford the full course of treatment?  ***(Circle all mentioned)*** | Shorten the course of treatment A  Offer deferred payment option B  Suggest treatment with lower cost  medicines C  Refer to public/ government facility ….. D  Other (Specify)________________ X  Don’t know/never happens Z |  |
| --- | --- | --- | --- |
| 254 | What do you do if the infant develops side effects to treatment?  ***(Circle all mentioned)*** | Discontinue treatment A  Refer to hospital B  Switch to alternate medicine C  Other (Specify)________________ X  Don’t know/never happens Z |  |
| 255 | What do you do if caregivers refuse to continue treatment?  ***(Circle all mentioned)*** | Counsel on importance of completing  treatment A  Refer B  Offer an alternate treatment C  Discontinue treatment D  Other (Specify)________________ X  Don’t know/ never happens Z |  |
| 256 | Under what circumstances, if any, do you decide to shorten the course of antibiotic treatment in a sick young infant?  ***(Circle all mentioned)*** | When child’s condition improves A  If caregivers cannot afford full course  ……………………………………...B  If child develops side effects C  If caregivers refuse to continue D  Other (Specify)________________ X  Never abbreviate course Y |  |
| 257 | What actions, if any, are taken for those who do not return for follow-up as expected?  ***(Circle all mentioned)*** | Phone family A  Send someone to find the family B  Other (Specify)________________ X  No action is taken Y |  |

**Section D. Observation of equipment and reference materials for management of sick young infants**

***During assessment and treatment of sick young infants, the service provider may have used the following medicines, equipment/ instruments and reference materials. Observe those, and in case they are not easily seen, ask the service provider, observe and then select ONE appropriate code for each item.***

| 258. Determine availability and functionality for each item of equipment listed below | | **Available and functioning** | **Available but not functional** | **Not available** |
| --- | --- | --- | --- | --- |
| 1 | Salter scale | 1 | 2 | 3 |
| 2 | Pan scale | 1 | 2 | 3 |
| 3 | Other infant scale (Specify) ______________ | 1 | 2 | 3 |
| 4 | Adult scale | 1 | 2 | 3 |
| 5 | Thermometer (digital) | 1 | 2 | 3 |
| 6 | Thermometer (other) | 1 | 2 | 3 |
| 7 | Stethoscope | 1 | 2 | 3 |
| 8 | Pulse oximeter | 1 | 2 | 3 |
| 9 | Timer/watch for counting respiratory rate | 1 | 2 | 3 |
| 10 | Cell phone | 1 | 2 | 3 |
| 11 | Injection syringe | 1 | 2 | 3 |

| 259. Determine availability for each item listed below | | | **Available** | **Available but not useable (expired)** | **Not available** |
| --- | --- | --- | --- | --- | --- |
| 1 | ORS sachets | | 1 | 2 | 3 |
| 2 | Zinc tablets | | 1 | 2 | 3 |
| 3 | Paracetamol | | 1 | 2 | 3 |
| 4 | Amoxycillin syrup/suspension | | 1 | 2 | 3 |
| 5 | Amoxycillin dispersible pediatric-dosed tablets | | 1 | 2 | 3 |
| 6 | Amoxycillin tablets/ capsules – 250mg (non-dispersible) | | 1 | 2 | 3 |
| 7 | Cotrimoxazole syrup/suspension or dispersible pediatric-dosed tablets | | 1 | 2 | 3 |
| 8 | Amox-clavulanate tablets (or suspension) | | 1 | 2 | 3 |
| 9 | Cefixime tablets (or suspension) | | 1 | 2 | 3 |
| 10 | Gentamycin injection (80mg/2cc amps) | | 1 | 2 | 3 |
| 1 | Ampicillin injection | | 1 | 2 | 3 |
| 12 | Cefuroxime injection | | 1 | 2 | 3 |
| 13 | Cefotaxime injection | | 1 | 2 | 3 |
| 14 | Ceftriaxone injection | | 1 | 2 | 3 |
| 15 | Insulin syringe for injectable antibiotics | | 1 | 2 | 3 |
| 16 | Insulin Syringe (0.5 ml) | | 1 | 2 | 3 |
| 17 | IV fluids | | 1 | 2 | 3 |
| 18 | IV line | | 1 | 2 | 3 |
| 260. Ask to see the following reference materials/job aids and other support materials. | | | **Available** | **Not available** |  |
| 1 | | IMNCI treatment guideline | 1 | 2 |  |
| 2 | | CIMS | 1 | 2 |  |
| 3 | | MIMS | 1 | 2 |  |
| 4 | | Other relevant reference materials (Specify) _______ | 1 | 2 |  |
| 5 | | Register for recording sick child cases | 1 | 2 |  |

**Thank the respondent for his/ her precious time and information and END the interview!!!!**

**Tool 2. Providers at private drug shops who only dispense treatment for sick young infants 0-2 months (i.e. not involved in treatment decisions; treatments dispensed based on prescription from a health worker / physician)**

**Section A: Background Characteristics**

I would like to ask you some general information about you.

| **S.No.** | **Questions** | **Responses** | **Go To** |
| --- | --- | --- | --- |
| 301 | Sex | Male 1  Female 2 |  |
| 302 | How old are you? | Completed years |  |
| 303 | What is your highest academic qualification, related to medical care? | Medical doctor 1  Nurse 2  ANM 3  Pharmacy assistant/Pharmacist 4  HA 5  CMA 6  No formal Qualification but have  worked as VHW/MCHW 7  No formal Qualification 8  Others (Specify)_______________96 |  |
| 304 | Have you ever received training on IMCI/IMNCI? | Yes 1  No 2 |  |
| 305 | Have you ever received training on CBNCP? | Yes 1  No 2 |  |
| 306 | How long have you been in private practice dispensing treatment for sick young infants (at this shop or another place)?  ***(Write in months***  ***If <1 month, write “000”)*** | Months……….................... |  |
| 307 | How long have you been working in this medicine shop?  ***(Write in months***  ***If <1 month, write “000”)*** | Months……….................... |  |
| 308 | What are the hours this shop is usually open?  ***(use 24 hh clock; hh:mm)*** | **a**) Open 24 hrs……………………0  **Hours Minutes**  b) Opening time :  c) Closing time : |  |
| 309 | How many days per week is this shop open? | Days |  |
| 310 | For how many hours per day are you (or another health worker) usually present at the shop? | Hours |  |
| 311 | Where do you work besides here?  ***(Circle all mentioned)*** | Government Health facility A  Other government sector B  Other private health sector facility C  Other private sector, non-healthcare D  Others (specify)________________X  Nowhere else Y |  |
| 312 | Is there usually another qualified health worker (at least CMA qualification) here when you’re away? | Yes 1  No 2 |  |

**Section B: Dispensing of Treatment for Sick Young Infants 0-2 months**

Now I would like to ask you some questions regarding dispensing the treatment to sick young infants 0-2months of age

| **S.No.** | **Questions** | **Responses** | **Go To** |
| --- | --- | --- | --- |
| 313 | About how many sick infants 0-2 months of age have you dispensed treatments for over the past 6 months?  ***(If > 995, write 995)*** | Estimated No. of cases ≤2 months cases………………………………….  Don't know 998 |  |
| 314 | About how many of these were 0-1 month of age?  ***(If > 995, write 995)*** | Estimated No. of cases ≤1months cases.……………………..  Don't know 998 |  |
| 315 | To about how many of them (0-2 month infants) did you dispense oral antibiotics over that period? | None of them 1  Some of them (i.e. less than half) 2  Most of them (i.e. more than half) 3  All of them 4  Don’t know……………………….98 | 317 |
| 316 | What specific oral antibiotic do you most often dispense for treatment of sick young infants 0-2 months old? | Amoxycillin 1  Cotrim 2  Amox-clavulanate 3  Azithromycin 4  Cefixime 5  Cefotaxime 6  Cefpodoxime 7  Other (Specify)________________ 96 |  |
| 317 | Over the past 6 months, about how many sick young infants 0-2 months old did you dispense injectable steroids (like dexamethasone)?  ***(If > 995, write 995)*** | None 0  No. given steroids  Don’t know 998 |  |
| 318 | Over the past 6 months, for about how many sick young infants 0-2 months old did you dispense injectable antibiotics?  ***(If > 995, write 995)*** | No. of ≤2 months given injectable  antibiotics  Don’t know 998  None……………………………….0 | 321 |
| 319 | About how many of these were 0-1 month of age?  ***(If > 995, write 995)*** | No. of ≤1 months given injectable  antibiotics  Don’t know 998  None……………………………….0 |  |
| 320 | What injectable antibiotics do (did) you most often dispense for treating sick young infants 0-2 months old?  ***(Probe ‘any other?’ and circle all mentioned)*** | Gentamycin A  Ampicillin B  Penicillin C  Ceftriaxone D  Cefuroxime E  Cefotaxime F  Other (Specify)_______________ X |  |

**Section C: Observation of Medicines**

***During assessment and treatment of sick young infants, the service provider may have used the following medicines. Observe those, and in case they are not easily seen, ask the service provider, observe and then select ONE appropriate code for each item.***

| 321. Determine availability for each item listed below | | **Available and usable** | **Available but not useable (expired)** | **Not available** |
| --- | --- | --- | --- | --- |
| 1 | ORS sachets | 1 | 2 | 3 |
| 2 | Zinc tablets | 1 | 2 | 3 |
| 3 | Paracetamol | 1 | 2 | 3 |
| 4 | Amoxycillin syrup/suspension or dispersible pediatric dosed tablets | 1 | 2 | 3 |
| 5 | Cotrimoxazole syrup/suspension or dispersible pediatric dosed tablets | 1 | 2 | 3 |
| 6 | Amox-clavulanate tablets | 1 | 2 | 3 |
| 7 | Cefixime tablets | 1 | 2 | 3 |
| 8 | Gentamycin injection | 1 | 2 | 3 |
| 9 | Ampicillin injection | 1 | 2 | 3 |
| 10 | Cefuroxime injection | 1 | 2 | 3 |
| 11 | Cefotaxime injection | 1 | 2 | 3 |
| 12 | Ceftriaxone injection | 1 | 2 | 3 |
| 13 | Insulin Syringe (1.0 ml) | 1 | 2 | 3 |
| 14 | IV fluids | 1 | 2 | 3 |
| 15 | IV line | 1 | 2 | 3 |

**Thank the respondent for his/ her precious time and information and END the interview!!!!**
